# Cholesterol reprograms glucose and lipid metabolism to promote proliferation in colon cancer cells

**DOI:** 10.1186/s40170-023-00315-1

**Published:** 2023-09-13

**Authors:** Shyamananda Singh Mayengbam, Abhijeet Singh, Himanshi Yaduvanshi, Firoz Khan Bhati, Bhavana Deshmukh, Dipti Athavale, Pranay L. Ramteke, Manoj Kumar Bhat

**Affiliations:** National Centre for Cell Science, Department of Biotechnology, Government of India, Savitribai Phule Pune University Campus, Ganeshkhind, Pune, 411 007 India

**Keywords:** LDLc, HDLc, Cholesterol, Glucose metabolism, Lipid metabolism, Colon cancer

## Abstract

Hypercholesterolemia is often correlated with obesity which is considered a risk factor for various cancers. With the growing population of hypercholesterolemic individuals, there is a need to understand the role of increased circulatory cholesterol or dietary cholesterol intake towards cancer etiology and pathology. Recently, abnormality in the blood cholesterol level of colon cancer patients has been reported. In the present study, we demonstrate that alteration in cholesterol levels (through a high-cholesterol or high-fat diet) increases the incidence of chemical carcinogen-induced colon polyp occurrence and tumor progression in mice. At the cellular level, low-density lipoprotein cholesterol (LDLc) and high-density lipoprotein cholesterol (HDLc) promote colon cancer cell proliferation by tuning the cellular glucose and lipid metabolism. Mechanistically, supplementation of LDLc or HDLc promotes cellular glucose uptake, and utilization, thereby, causing an increase in lactate production by colon cancer cells. Moreover, LDLc or HDLc upregulates aerobic glycolysis, causing an increase in total ATP production through glycolysis, and a decrease in ATP generation by OXPHOS. Interestingly, the shift in the metabolic status towards a more glycolytic phenotype upon the availability of cholesterol supports rapid cell proliferation. Additionally, an alteration in the expression of the molecules involved in cholesterol uptake along with the increase in lipid and cholesterol accumulation was observed in cells supplemented with LDLc or HDLc. These results indicate that colon cancer cells directly utilize the cholesterol associated with LDLc or HDLc. Moreover, targeting glucose metabolism through LDH inhibitor (oxamate) drastically abrogates the cellular proliferation induced by LDLc or HDLc. Collectively, we illustrate the vital role of cholesterol in regulating the cellular glucose and lipid metabolism of cancer cells and its direct effect on the colon tumorigenesis.

**Graphical Abstract:**

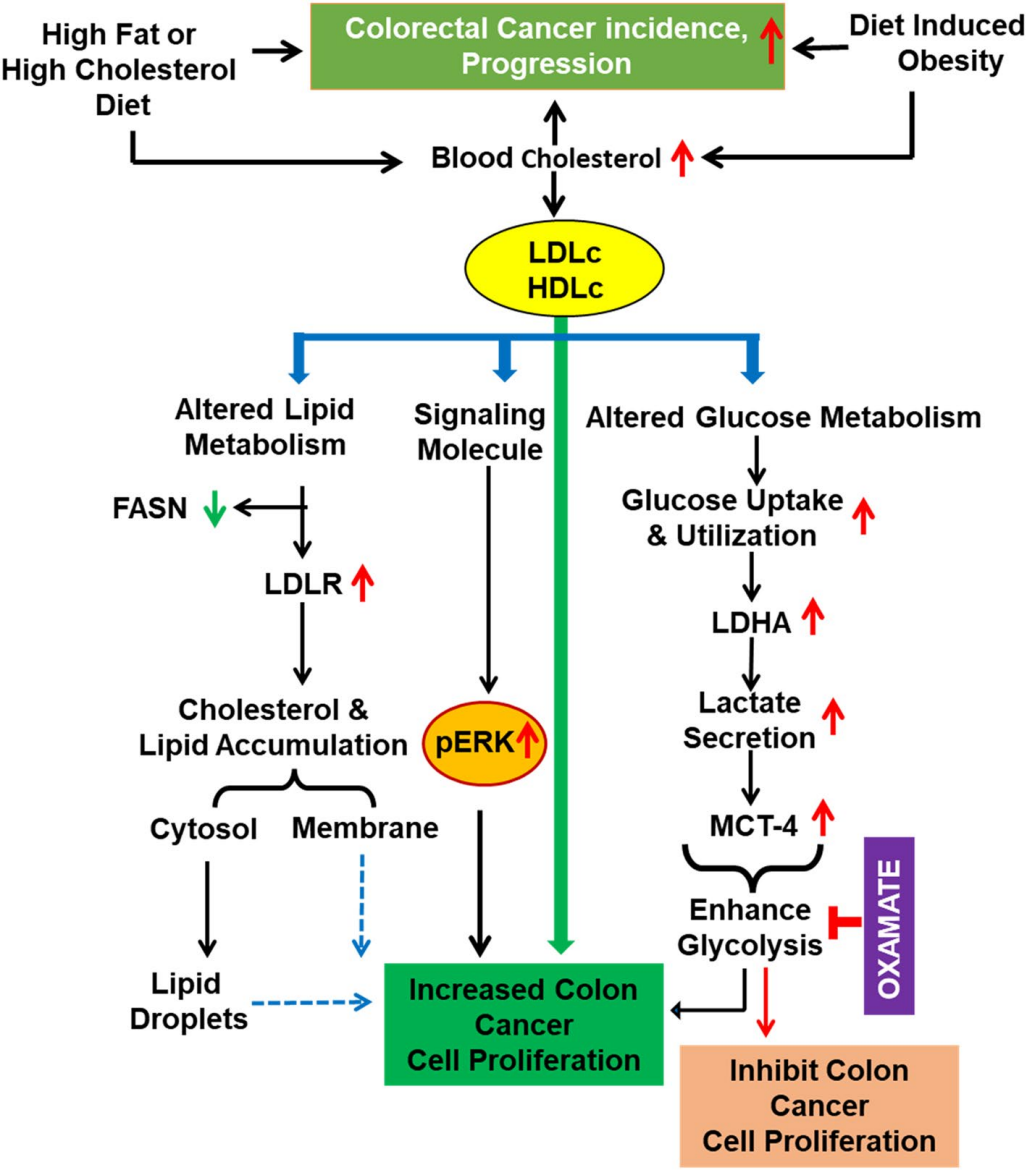

**Supplementary Information:**

The online version contains supplementary material available at 10.1186/s40170-023-00315-1.

## Background

The number of hypercholesterolemic individuals, dependent or independent of obesity, is increasing rapidly worldwide [1]. Though the exact percentage of the non-obese dyslipidemic population is unclear, reports suggest that its prevalence ranges from 10 to 27% [2]. An increase in the level of total blood cholesterol, circulatory low-density lipoprotein cholesterol (LDLc), triglycerides, and a decrease in high-density lipoprotein cholesterol (HDLc) levels are primarily associated with overweight and obesity [3]. Obesity is an established risk factor for colon cancer with a relative risk of approx. 1.5 to 2.0 in men, 1.2 to 1.5 in women [4, 5], and worsen the prognosis of cancer patients [6]. Earlier findings from our group have demonstrated the effect of obesity on promoting colon cancer initiation, and melanoma tumor progression in mouse models [7, 8]. However, concrete evidence through which hypercholesterolemia influences the risk of cancer, and tumorigenesis is still unclear. Cholesterol could likely be a major contributing factor in increasing cancer risk, promoting cancer cell proliferation, and worsening prognosis in obesity-influenced cancers.

In the recent past, clinical and preclinical studies have highlighted a possible link between high-cholesterol diet or hypercholesterolemia, with colon cancer [9, 10]. Moreover, alteration in blood cholesterol levels in colon cancer patients is also a frequently observed phenomenon. Clinical studies have reported a decrease in the blood cholesterol level in colon cancer patients, which inversely correlates with the increased grade of polyps/tumors [11–13]. Preclinical studies in colon and breast cancer have also reported the functionality of cholesterol in regulating various signaling pathways through the phosphorylation of AKT and ERK [14, 15]. In another study, it has been shown that cholesterol functions as a mitogen for intestinal stem cells (ISC) and promotes the proliferation of progenitor cells or intestinal crypt [10, 15].

In many cancers, aberrant lipid metabolism is a common phenomenon, leading to upregulation in cholesterol uptake or biosynthesis to sustain rapid cell proliferation [16].

In normal cells, the biological functions of cholesterol are diverse. It is an integral structural component of the cell membrane and is also involved in the biosynthesis of hormones and vitamins [16]. Recent studies have demonstrated that the use of statin a cholesterol-lowering agent is preventive towards cardiovascular disease, but it increases the risk of diabetes. Also, in familial hypercholesterolemic patients, elevated serum LDLc is linked to a decrease in risk of type 2 diabetes mellitus [17], suggestive of a correlation between LDLc and alteration in glucose metabolism. Moreover, HDLc can also interfere with the cellular glucose metabolism in normal adipocytes (3T3-L1) as well as in skeletal muscle cells [18, 19]. Another recent study by Broadfield et al. reported that the glucose metabolism of non-transformed normal hepatocyte cells can be altered by fat [20]. These findings are indicative of a correlation between lipid/fat components (i.e., LDLc or HDLc) towards regulating glucose metabolism in normal cells. However, in most cancers, cellular glucose metabolism is reprogrammed towards a more glycolytic phenotype, thereby metabolizing the available glucose primarily through aerobic glycolysis [21]. The increase in the rate of aerobic glycolysis is considered a characteristic feature of aggressive cancers [22].

The present work primarily focuses on understanding the role of cholesterol in supporting cancer cell proliferation, reprogramming of cellular metabolism and tumorigenesis in colon. Recently, it has been reported that cholesterol through the activation of the MAPK pathway promotes the proliferation of colon cancer cells [15]. Interestingly, the effect of cholesterol has thus by far not been linked to alteration in cellular glucose or lipid metabolism of cancer cells.

Through in vitro and in vivo experimentation, we have delineated the role of cholesterol in supporting colon tumorigenesis, progression, and cellular proliferation. Results presented herein suggest that LDLc or HDLc facilitate the rapid proliferation of cancer cells through alteration in glucose and lipid metabolism. Furthermore, this study highlights that targeting upregulated glucose metabolism by utilizing an LDH enzyme inhibitor (oxamate) can significantly retard the cholesterol-mediated proliferation of colon cancer cells. Collectively, these findings may have clinical relevance in the context of cancer influenced by cholesterol.

## Materials and methods

### Cell lines and culture conditions

Murine colon cancer cell line, MC-38, was generously gifted by Dr. Ruben Hernandez, CIMA, Spain. Human colon cancer cell lines, HCT-116 p53 + / + (HCT-116) and HCT-116 p53 − / − were kindly gifted by Dr. Bert Vogelstein, John Hopkins University, USA. HCT-15, HT-29, and Caco-2 were procured from the in-house cell repository of the National Centre for Cell Science (NCCS, Pune, India). Cells were cultured in Dulbecco’s modified Eagle’s medium (DMEM), supplemented with 10% fetal bovine serum (FBS) (Gibco, NY, USA), 100 μg/ml streptomycin, and 100 U/ml penicillin (Invitrogen Life Technologies, CA, USA) and maintained in temperature-controlled (37 °C), humidified CO_2_ (5%) incubator (Thermo Fisher Scientific, OH, USA).

### Antibodies and chemicals

Antibody against LDLR (Cat # ab-30532),  MCT-1 (Cat # ab-90582), MCT-3 (Cat # ab-60333) and SR-B1 (Cat # 217318) were purchased from Abcam (Cambridge, UK); antibodies against FASN (Cat # sc-55580), MCT-4 (Cat # sc-50329), MCT-2 (Cat # sc-166925), LDHA (Cat # sc-137244), LXRα/β (Cat # sc-377260), ACAT-1 (Cat # sc-517387), ACAT-2 (Cat # sc-293307), phospho-ERK (Tyr 204) (Cat # sc-7383), ERK (Cat # sc-154), Cyclin A (Cat # sc-751), CDK 2 (Cat # sc-163), Cyclin E (Cat # sc-198), β-tubulin (Cat # sc-9104), GAPDH (Cat # sc-20357), β-actin (Cat # sc-1615), and HRP conjugated secondary antibodies: anti-rabbit (Cat # sc-2030), anti-mouse (Cat # sc-2031), anti-goat (Cat # sc-2033) were purchased from Santa Cruz Biotechnology (CA, USA). Human LDL cholesterol (Cat # 360–10) and human HDL cholesterol (Cat # 361–10) were purchased from Lee Biosolution (MO, USA); Water soluble-free cholesterol and Dextran sodium sulfate (DSS) (Cat # 160110) were purchased from MP Biomedicals (CA, USA). Azoxymethane (AOM) (Cat # A5486), 2-Deoxy-D-glucose (2-DG) (Cat # D8375) and Oxamate (Cat # O2751) were purchased from Sigma Aldrich (MO, USA).

### Animal experiments and diets

Male C57BL/6 J mice (6–8 weeks old) were procured from the Experimental Animal Facility (EAF) of the National Centre for Cell Science (NCCS), Pune, India. All mice were housed and maintained in animal quarters under controlled environmental conditions of 22 ± 2 °C with a light and dark cycle of 12 h, along with free access to clean drinking water and standard rodent pellet food ad libitum, unless otherwise mentioned. Normal diet (ND, 5% fat) was purchased from Amrut Laboratory, Pune, India; high-cholesterol diet (HCD, 5% fat, 1.25% cholesterol with 0.5% sodium cholate), and high-fat diet (HFD, 24% fat) was procured from VRK Nutrition Pvt. Ltd., Pune, India.

#### Development of diet-induced obesity and hypercholesterolemic mice model

Diet-induced obese mice model was developed by feeding animals with a high-fat diet, supplemented with groundnut and coconut as described previously [23]. Hypercholesterolemic mice were developed by feeding them with high-cholesterol diets. In each experiment, mice were divided into various groups depending on the types of diet provided to them (normal diet—ND group, high-fat diet—HFD group, and high-cholesterol diet—HCD group), till the completion of the experiment. Body weight and serum chemistry analysis were performed to verify the status of obesity-associated changes and hypercholesterolemic phenotype in both HFD and HCD groups in comparison to the ND group.

#### Development of AOM/DSS-induced colon cancer initiation

Mice were randomly divided into ND (*n* = 15), HCD (*n* = 15), and HFD (*n* = 15) groups and fed on their respective diets. After 2 months on diet, mice were subjected to AOM/DSS treatment for chemically induced colon polyp formation as described previously [24]. Mice were sacrificed within 15–20 days of the completion of the AOM/DSS cycles, and colon tissues were taken out for analysis of the polyp’s development. In each group, the incidence of polyp occurrence (ratio of animals having polyps to the total number of animals in each group), the number of polyps per colon, polyp diameter, and colon length were recorded. Colon tissues including polyps were preserved at − 80ºC as frozen tissue for further investigation.

#### Isograft tumor development

For the isograft model of tumor development, two different sets of experiments were performed. In the first experiment, mice were randomized into two groups, the ND group & HFD group. In the second experiment, mice were grouped into ND & HCD groups. Mice were kept on their respective diet for 4 to 6 months prior to the implantation of MC-38 cells. Mice exhibiting normal (from ND group, (*n* = 5)), obese (from HFD group, (*n* = 5)), and hypercholesterolemic (from HCD group, (*n* = 5)) phenotypes were subcutaneously implanted with MC-38 cells (1 × 10^6^ cells /100 µl in PBS) into the right flank. Once the tumor size became palpable, its size was monitored in each group. The length and width of the tumor were recorded on every third day and tumor volume was calculated by using the standard formula: 0.52 × length × width^2^. At the end of the experiment, mice were sacrificed by CO_2_ euthanasia. Tumors were excised, weighed, and the samples were preserved at − 80 °C.

All animal experiments were performed as per the requirement and guidelines of the Committee for the Purpose of Control and Supervision of Experiments on Animals (CPCSEA), Government of India, and after getting the permission of the Institutional Animal Ethics Committee (IAEC) (IAEC/2018/B-335).

### Serum biochemical analysis

Blood from ND, HFD, and HCD mice was collected in vacutainer serum collecting tubes through the retro-orbital puncture. The serum was separated by centrifuging the blood at 6000 rpm for 5 min at 4 °C for biochemical analysis. Serum cholesterol (Cat # TK41021), triglyceride (Cat # MX41031), and glucose (Cat # MD41011) estimation were performed through a standard estimation kit purchased from Spinreact (Girona, Spain). Blood glucose was also measured by using an Accu-check analyzer from Roche Diagnostics (Mannheim, Germany) as per manufacturer protocol. Levels of serum adiponectin and leptin were estimated by using mouse-specific adiponectin/Acrp30 immunoassay ELISA kits, R&D System (Cat # MRP300, MN, USA), and leptin (mouse) EIA kit, Enzo life science (Cat # 900-019A), as per manufacturer’s protocol. All the experiments were performed in the pooled serum sample of each group (*n* = 4).

### Cholesterol (LDLc or HDLc or free cholesterol) / inhibitors treatment

Appropriate numbers of cells (1.5–3 × 10^5^ cells/well) were seeded in 12- or 6-well or 35-mm cell culture plates and allowed to grow for 24 h. Cells were then serum starved for 12–16 h, followed by treatment with varying concentrations of LDLc or HDLc or free cholesterol or vehicle (water/PBS) in DMEM containing 1% FBS for different time points (12 or 24 or 48 or 72 h) as per experimental requirements. For inhibitor experiments, cells were treated with oxamate (15 mM) in the presence or absence of LDLc or HDLc for different time points as per requirements.

### Long-term colony formation and crystal violet cell survival assay

Cells were seeded (200 to 500 cells/well) in 24- or 12-well culture plates. Spent media were removed and replaced with fresh DMEM (1% FBS) media containing vehicle or LDLc or HDLc. These were then allowed to grow in their respective media for another 12 to 15 days with media change on every alternate day. Subsequently, cells were fixed and stained with crystal violet (Cat # C-6158, Sigma) as described earlier [25]. Cell survival assay was done by seeding appropriate numbers of cells in 12 or 24-well plates and allowed them to grow up to 40–50% confluency. They were treated with LDLc or HDLc in 1% FBS-containing media with or without oxamate for 72 h. Cells were then fixed, stained with crystal violet, and analyzed as described previously [25].

### Cell cycle analysis

Cells were seeded (3 × 10^5^ cells/well) in the 35-mm cell culture plate. After 24 h of incubation, cells were serum starved for another 12–16 h followed by treatment with vehicle or LDLc or HDLc in the presence or absence of drugs or inhibitors for another 48 h. These were then trypsinized, harvested, and processed for cell cycle analysis as described previously [25]. Cells (10,000 events each) were then analyzed using the flow cytometer (BD Canto II). Data generated was examined in FlowJo software (OR, USA), and the percentage of cell population in different phases of cell cycles was determined.

### Immunoblotting and immunofluorescence

Immunoblotting of molecules from the lysate of tumor tissues and cells pre-treated with vehicle or LDLc or HDLc was performed as per the protocol described previously [23]. For immunofluorescence imaging, cells were plated in 24- or 12-well plates containing sterile round glass coverslips and allowed to grow for 24 h followed by serum starvation for 12 h. After 48 h of LDLc and HDLc treatment, cells were processed for immunofluorescence imaging as described previously [23] and observed under a Leica SP5 II confocal microscope (Leica Microsystems, Germany). Images were analyzed through LAS image analysis software. MCT-4, MCT-1, MCT-2, MCT-3, LDHA, pERK, ERK, LDLR, ACAT-1, ACAT-2, FASN, LXRα/β, SR-B1, Cyclin E, Cyclin A, CDK 2, GAPDH, β-actin, and β-tubulin were probed for immunoblotting and MCT-4 for immunofluorescent imaging.

### Nile Red staining for lipid accumulation

Cells pre-treated (48 h) with vehicle/LDLc/HDLc were fixed with 3.7% PFA for 15 min followed by two times PBS washing and then incubated in PBS containing Nile red (1 µg/ml) for 10 min at 37 °C. After washing with PBS, the sample was mounted in a mounting medium containing DAPI. Images were acquired in Leica confocal microscope and analysis was done through LAS image analysis software.

### Cholesterol estimation in cell lysate and spent media

Cells pre-treated with vehicle or LDLc or HDLc were washed with chilled PBS, lysed with 100 µl of 1% Triton X-100 solution, and incubated for 30 min in ice followed by centrifugation at 12,000 rpm for 30 min. Cholesterol estimation was done from the whole cell lysate and the values were normalized with total protein concentration. Spent media from the cells treated with vehicle or LDLc or HDLc were concentrated in a speed vacuum concentrator (SC110A speed vac plus, Thermo Savant). Cholesterol, LDLc, and HDLc were estimated as per the protocols of respective kits.

### Fluorescence-labelled glucose uptake

Cells treated with vehicle or LDLc or HDLc were washed with sterile PBS thrice followed by the addition of glucose-free medium in each well. Cells were then treated with 20 µM of fluorescent glucose analog 2-NBDG (2-(N-(7-Nitrobenz-2-oxa-1,3-diazol-4-yl) Amino)-2-Deoxyglucose) (Cat # N13195) for 30 min at 37 °C, with or without LDLc or HDLc followed by three times washing with PBS. Cells were harvested and processed for FACS analysis. Data sets were acquired (10,000 events each) in BD FACS Canto II, and analysis was done in De novo FCS Express software (CA, USA).

### Glucose and Lactate estimation in spent media

HCT-116 and HCT-15 (3 × 10^5^ cells/well) cells were seeded in 12-well cell culture plates and allowed to grow for 24 h, followed by serum starvation for 12 h. Media were replaced with fresh DMEM (1% FBS) containing vehicle or LDLc or HDLc or free cholesterol for another 24 h. Glucose and lactate present in spent media were quantified by glucose and lactate estimation kit Spinreact (Cat # 41,013 and Cat # 1,001,330 respectively, Girona, Spain) as per the manufacturer’s protocol.

### Lactate dehydrogenase enzyme assay

Cells pre-treated with vehicle or LDLc or HDLc were homogenized in ice-cold homogenization buffer (20 mM Tris–HCl, pH 7.5, 250 mM Sucrose, 5 mM EDTA, 80 mM KCl, 4 mM MgCl_2_, 1 × Protease K) as described previously [26]. Homogenates were centrifuged at 12,000 rpm for 15 min at 4 °C followed by centrifugation of the resulting supernatant at 100,000 rpm for 60 min in the ultracentrifuge. The cytosolic fractions obtained were used for the LDH enzyme activity assay, and 15 μL of the cytosolic fraction was used in 150 μL of total reaction volume. LDH activity assay was performed by using the kit (Cat # 1,001,260; Spinreact, Girona, Spain) as per manufacturer’s protocol. All the values were normalized with protein concentration. Absorbance was recorded at 340 nm every 1 min for at least 5 min (5 times) using a spectrophotometer.

### Mitochondrial density

For measurement of mitochondrial density, cells were washed with PBS and resuspended in PBS containing 50 nM Mito Tracker Red FM dye (Cat # 31838W, Life Technologies, MA, USA) for 30 min at 37 °C. Cells were then washed with PBS thrice and harvested for FACS analysis. Data sets were acquired (10,000 cells) in Canto BDII flow cytometer and analysis was done in de novo FCS Express software (CA, USA).

### Glyco stress, mitostress, and real-time ATP rate assay

Cells (3 × 10^4^ to 4 × 10^4^ cells/well) were plated into 24-well XF Cell culture plates (Cat # 100,777–004; Agilent Technologies, CA, USA) and allowed to grow overnight in a 5% CO_2_ incubator at 37 °C. Cells were then treated with vehicle or LDLc or HDLc or free cholesterol or inhibitor for 12 h in 1% FBS-containing media and assays were performed. After treatment, cells were washed with their respective assay medium and incubated in a humidified non-CO_2_ incubator at 37 °C for 30 min with 500 µl of assay medium (XF base or DMEM medium) and the following assays were performed.

#### Glyco stress assay

Glyco stress assay was performed in Agilent XF base medium (Cat # 102,353–100) as per protocol provided by Agilent Seahorse XF Glycolysis Stress Test Kit user guide. Four ports (Port A, B, C, D) were utilized to inject various compounds or inhibitors into each well. LDLc or HDLc or inhibitors or vehicle was injected through port A and allowed to incubate for 45 min, followed by injection of glucose, oligomycin, and 2-Deoxy-D-glucose (2-DG) in ports B, C, and D, respectively in each well. Extracellular acidification rate (ECAR) was measured by using the Seahorse XFe24 analyzer, Agilent (CA, USA).

#### Mitostress assay for mitochondrial function

Experiments were performed in Agilent XF base media (102,353–100) as per protocol provided by the Agilent Seahorse XF Cell Mito Stress Test Kit user guide. LDLc or HDLc or free cholesterol or inhibitors or vehicle was injected through port A and allowed to incubate for 45–50 min, followed by injection of oligomycin, 0.5 µM carbonyl cyanide-4(trifluoromethoxy) phenylhydrazone (FCCP), and rotenone & antimycin A in ports B, C, and D respectively in each well. Oxygen consumption rate (OCR) was measured by using Seahorse XFe24 analyzer Agilent (CA, USA).

### Real-time ATP rate assay

Experiments were performed in Agilent XF DMEM medium (103,576–100) as per protocol provided by Agilent Seahorse XF real-time ATP rate assay kit user guide. LDLc or HDLc or vehicle was injected through port A and allowed to incubate for 45–50 min, followed by injection of oligomycin, and rotenone & antimycin A in ports B, C, and D respectively in each well. OCR and ECAR were measured by using a Seahorse XFe24 analyzer, Agilent (CA, USA). Data was analyzed in seahorse wave desktop software (CA, USA).

### Wound healing scratch assay

Cells were seeded in 6-well plates and allowed to become 80–90% confluent followed by serum starvation for 24 h in the serum-free media. Thereafter, scratches were made on the plates by using a 200-μl pipet tip followed by three times washing with PBS. Cells were cultured in 1% FBS media containing 50 μg/ml HDLc or 50 μg/ml LDLc, and untreated vehicle control. Subsequently, images were captured at different time point (0, 6, and 18 h) using DP-71 Olympus camera (Shinjuku-ku Tokyo, Japan). Images were analyzed using ImageJ plugin Wound Healing Size Tool [27]. Experiment was performed twice and representative images along with bar graph from an experiment are shown.

### Kaplan–Meier plotter analysis

The prognostic values of LDLR (Affymetrix ID: 202068_s_at), LDHA (Affymetrix ID: 200650_s_at), FASN (Affymetrix ID: 212218_s_at), and MCT-4 (Affymetrix ID: 202856_s_at) mRNA levels in colon cancer were analyzed using KM plotter. For all Kaplan–Meier plots, the KM Plotter “auto select best cut-off” option was used for stratification of data. Plots were generated using multiple options such as the following: survival (overall survival (OS), relapse-free survival (RFS), and post progression survival (PPS)). Also, the data was analyzed from different stages of tumor (I–IV) and on the basis of gender (male and female). The plots generated mentioning hazard ratio (HR) and log rank *p* values are presented. Kaplan–Meier plotter gene expression data, OS, RPS, and PPS information are downloaded from GEO (Gene Expression Omnibus), EGA (European Genome-phenome Archive), and TCGA (The Cancer Genome Atlas). Datasets used for survival analysis includes GSE12945, GSE13294, GSE14333, GSE143985, GSE17538, GSE18088, GSE26682, GSE26906, GSE30540, GSE31595, GSE33114, GSE34489, GSE37892, GSE38832, GSE39582, and GSE41258 [28]. Additionally, LDLR, LDHA, MCT-4, and FASN gene expression in normal, tumor, and metastatic colon tissues were generated using TNMplot.com [29].

### Statistical analysis

Statistical analysis (Student’s 2-tailed unpaired *t*-test and one-way or two-way ANOVA) was performed by using Graph Pad Prism 8 Software for comparison of different groups. All data points were represented as the mean ± standard deviation (S.D). In majorities of the in vitro experiments, bars represent experimental variations within the wells, unless otherwise mentioned. The values of *P* < 0.05 were considered statistically significant (*P < 0.05, **P < 0.01, ***P < 0.001).

## Results

### High-cholesterol diet and high-fat diet increase carcinogen-induced colon cancer incidence and isograft tumor progression in mice

**Fig. 1:**
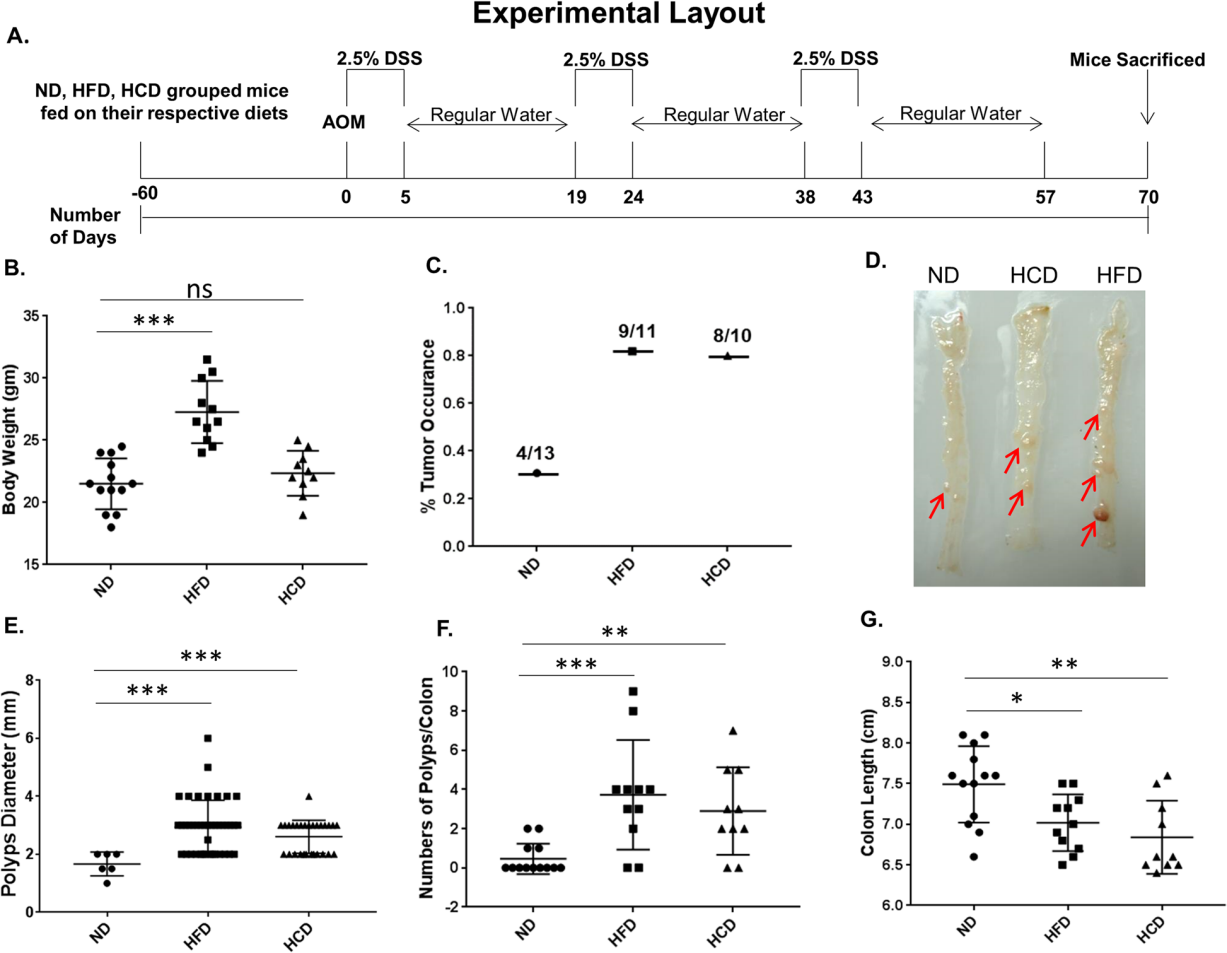
AOM/DSS-induced colorectal carcinogenesis in ND-, HFD-, and HCD-fed C57BL/6J mice. Chemically induced colon cancer was developed by injecting AOM (10 mg/kg body weight) and followed by three cycles of DSS (2.5%) in drinking water as mentioned in the methods section. **A** Experimental design for AOM/DSS-induced colorectal carcinogenesis, In ND-, HFD-, and HCD-fed mice. **B** Body weight recorded at the end of the experiment. **C** Percentage of polyp’s occurrence. **D** Picture of colon-bearing polyps. **E** Differences in polyp’s diameter. **F** No. of polyps per colon in each group. **G** Colon length

**Fig. 2:**
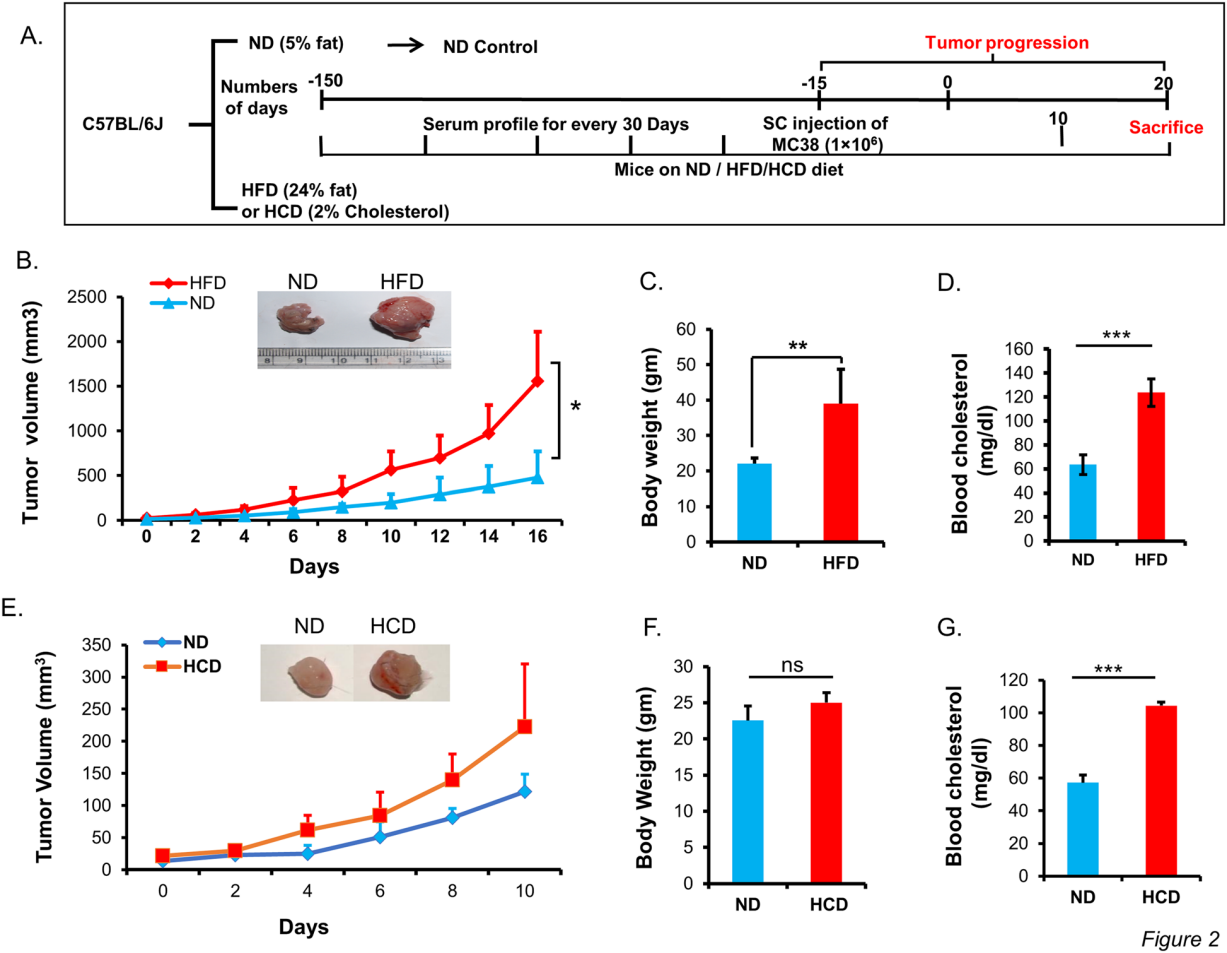
MC-38 isograft tumor induction in ND-, HFD-, and HCD-fed C57BL/6 J mice. Mice fed on different diets were subcutaneously implanted with MC-38 cells. **A** Experimental setup for MC-38 tumor progression in the ND-, HFD-, and HCD-fed mice. In ND and HFD mice, **B** tumor progression. **C** Mice body weight recorded at the end of the experiment. **D** Serum cholesterol level during feeding of diets, i.e., before MC-38 isograft implantation. In ND and HCD mice, **E** tumor progression and **F** difference in body weight recorded before the termination of the experiment. **G** Serum cholesterol level measured during feeding of diets, i.e., before MC-38 isograft implantation

Studies from our group and others have demonstrated the role of obesity in supporting rapid cancer progression. Earlier we reported that in AOM/DSS chemical carcinogen model the incidence of cancerous polyp formation was higher in diet-induced obese mice [8]. To evaluate specifically the role of cholesterol in colon cancer initiation and progression; AOM/DSS-mediated polyps’ formation, as well as MC-38 isograft-induced tumor progression models, were used. A comparative analysis for cancer incidences and tumorigenesis of the colon was studied in normal, hypercholesterolemic, and obese mice. In HCD mice, circulatory serum cholesterol levels were increased without any significant change in body weight and serum leptin level (i.e., hypercholesterolemic lean mice). Whereas in the HFD group, mice show a significant gain in body weight, along with an increase in serum cholesterol and leptin level (Figs. 1B and 2C-G; Supplementary table 1). These parameters indicate that HFD mice are of obese phenotype with altered serum biochemical parameters whereas HCD mice are non-obese and hypercholesterolemic and do not exhibit significant alteration in serum biochemistry.

AOM/DSS-induced carcinogenesis experiments were performed in ND, HFD, and HCD mice as per the experimental layout indicated in Fig. 1A. Results indicate that the percentage of polyp occurrence was much higher in HFD (9 out of 11, 81% occurrence) and HCD (8 out of 10, 80% occurrence) as compared to ND (4 out of 13, 30.95% occurrence) (Fig. 1C). The diameter of polyps was also comparatively more in HFD (2.93 mm) and HCD (2.607 mm) in comparison to ND (1.66 mm) (Fig. 1D, E). The number of polyps per colon was more in HFD (average 3.7 polyps/colon) and HCD (average 2.9 polyps/colon) as compared to ND (average 0.43 polyps/colon) (Fig. 1D, F). Additionally, the colon length in HFD and HCD mice was also shorter in comparison to ND mice (Fig. 1G). Taken together, it was observed that in HCD and HFD mice the incidence of polyp formation in the colon was comparable. However, the numbers of polyps per colon and polyps’ size were relatively greater in HFD as compared to HCD. It is likely that the differences observed could be due to factors particularly associated with obesity, in addition to cholesterol.

To investigate the effect of diet-induced hypercholesterolemia and obesity on colon cancer progression, the growth of iso-grafted MC-38 tumors was monitored in ND and HFD mice as per the experimental design shown in Fig. 2A. The average tumor volume in HFD mice was 1556.37 mm^3^ as compared to 476.75 mm^3^ in ND mice at identical time points, i.e., tumor weight is 3 folds greater in HFD (Fig. 2B). The result indicates that tumor progression in HFD mice was much faster in comparison to ND mice. Similarly in another set of experiments with ND and HCD, larger tumor size in the HCD mice group was observed as compared to the ND mice group, though the difference between the groups was not significant (Fig. 2E).

Collectively, in vivo, experimental results demonstrate that hypercholesterolemia (triggered by high-cholesterol diet or diet-induced obesity) can be a major contributing factor in increasing AOM/DSS-induced colon cancer incidence as well as in promoting tumor progression. Irrespective of body weight, high cholesterol levels in the blood or high cholesterol intake may have a positive impact on colon cancer incidence and progression. Herein, the result indicates that the increase in the incidence of polyp formation, larger polyps’ size, and faster tumor progression in diet-induced obesity and hypercholesterolemia can also be attributed partly due to an increase in blood cholesterol level or an increase in dietary cholesterol intake, which directly or indirectly affects the colonic tissue.

### LDLc and HDLc promote the proliferation and migration of cancer cells

**Fig. 3:**
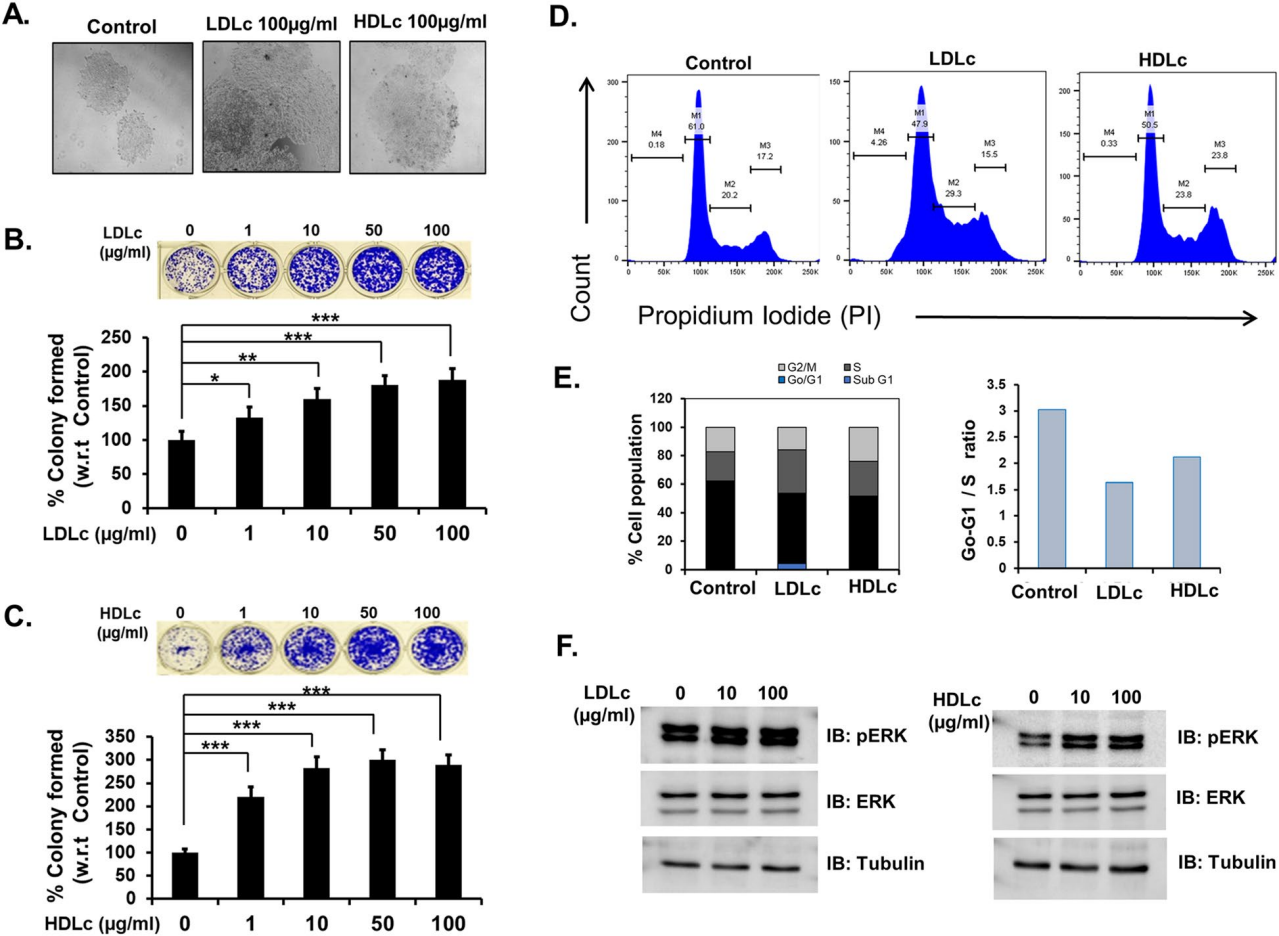
Effect of LDLc and HDLc supplementation on proliferation, cell cycle, and changes in signaling intermediate in colon cancer cells. HCT-116 cells were cultured in different concentrations of LDLc or HDLc, for different time points thereafter colony formation, cell cycle, and immunoblot analysis were performed as per the protocol mentioned in the methods. **A** Images of the colony were taken in a × 10 phase contrast microscope on the 12th day before staining with 0.05% crystal. **B**,**C** Images showing crystal violet-stained colonies of HCT-116 cells with or without LDLc, and HDLc treatment along with a bar graph representing the percent colony formed with respect to untreated controls. Colonies were stained with 0.05% crystal violet and images were captured in an Olympus DSLR camera and quantification was done using ImageJ software. Experiments were done in triplicate and performed twice. **D**,**E** Cell cycle analysis of HCT-116 cells treated with or without 50 µg/ml LDLc or HDLc for 48 h. **D** Histogram showing different phases of the cell cycle, and **E** bar graphs representing % cell population in different phases of the cell cycle, and the ratio of Go-G1/S-phase cell population with or without LDLc or HDLc treatment. Experiments were performed twice. **F** Immunoblot analysis of pERK and ERK protein expression was evaluated from the whole cell lysate of HCT-116 cells after treatment with different concentrations of LDLc or HDLc or vehicle control. Tubulin was used as a loading control. The experiment was performed twice

In vivo, studies indicate the involvement of cholesterol in supporting the initiation of cancerous polys formation in the colon as well as tumorigenesis. Subsequently, the effect of cholesterol on the growth of HCT-116, HCT-116 p53 − / − , HCT-15, Caco-2, and HT-29 cells was analyzed by long-term colony formation or MTT assay. Cells were cultured in media containing varying concentrations of human LDLc or HDLc. A significant increase in the proliferation of HCT-116 cells was observed by MTT and colony formation assays upon LDLc or HDLc treatment, in a dose-dependent manner (Fig. 3A–C; Supplementary Fig. 1 G-H). Cells treated with LDLc or HDLc form a greater number of colonies, which are also larger in size as compared to untreated/vehicle control (Fig. 3A–C). Similar results were also observed in HCT-116 p53 − / − , HCT-15, Caco-2, and HT-29 cells (Supplementary Fig. 1 A-F). Additionally, migration assay was performed to check the effect of cholesterol on colon cancer cell motility. It was observed that LDLc and HDLc increases the migration of HCT-116 cells (Supplementary Fig. 1I). Together, these results indicate that LDLc and HDLc promote the proliferation of colon cancer cells irrespective of their genetic background. Moreover, in LDLc or HDLc-induced proliferation, p53 status in cells does not play any significant role.

As the treatment of LDLc or HDLc promotes cell proliferation, the distribution of cells in various phases of the cell cycle with or without LDLc or HDLc treatment was also analyzed in HCT-116 cells. An increase in the percentage of cell population in S-phase and G2/M-phase along with the decrease in Go/G1-phase was detected in both LDLc- and HDLc-treated conditions as compared to untreated control (Fig. 3D). Moreover, the ratio of Go/G1 to S-phase was also decreased in LDLc- and HDLc-treated conditions, suggestive of cells being inclined towards a more proliferative state in treated conditions (Fig. 3E). Additionally, by immunoblot analysis upregulated expression of cyclin A, CDK 2, and cyclin E were detected in HCT-116 cells treated with LDLc or HDLc (Supplementary Fig. 9A). Interestingly, earlier studies in prostate cancer cells also suggest a possible correlation between cholesterol and cell cycle, in which an increase in cyclin E level along with the enhanced accumulation of cholesterol inside the nucleus and the cytoplasm was reported [30]. From these results, it is apparent that both LDLc and HDLc play an important role in regulating the cell cycle process which contributes to the rapid proliferation of cancer cells.

### HDLc and LDLc promote the phosphorylation of ERK

Earlier studies have reported the role of cholesterol in regulating various signaling pathways involved in cancer cell proliferation. Interestingly, in HCT-116 and HCT-15 cells, supplementation of LDLc or HDLc promotes the phosphorylation of ERK (Fig. 3F; Supplementary Fig. 4), which is in agreement with the previous findings, in different cancers [14]. These results validate that the presence of LDLc or HDLc can directly or indirectly regulate ERK-dependent signaling pathways for supporting colon cancer cell proliferation.

### LDLc and HDLc enhance lipid accumulation inside the cells

In vivo and in vitro experimental results indicated the involvement of cholesterol in supporting colon cancer initiation, tumor progression, and cellular proliferation, which is likely to be associated with a change in metabolic status (considered a hallmark of cancer). Therefore, the effect of LDLc and HDLc on the metabolic adaptability of colon cancer cells was evaluated.

**Fig. 4:**
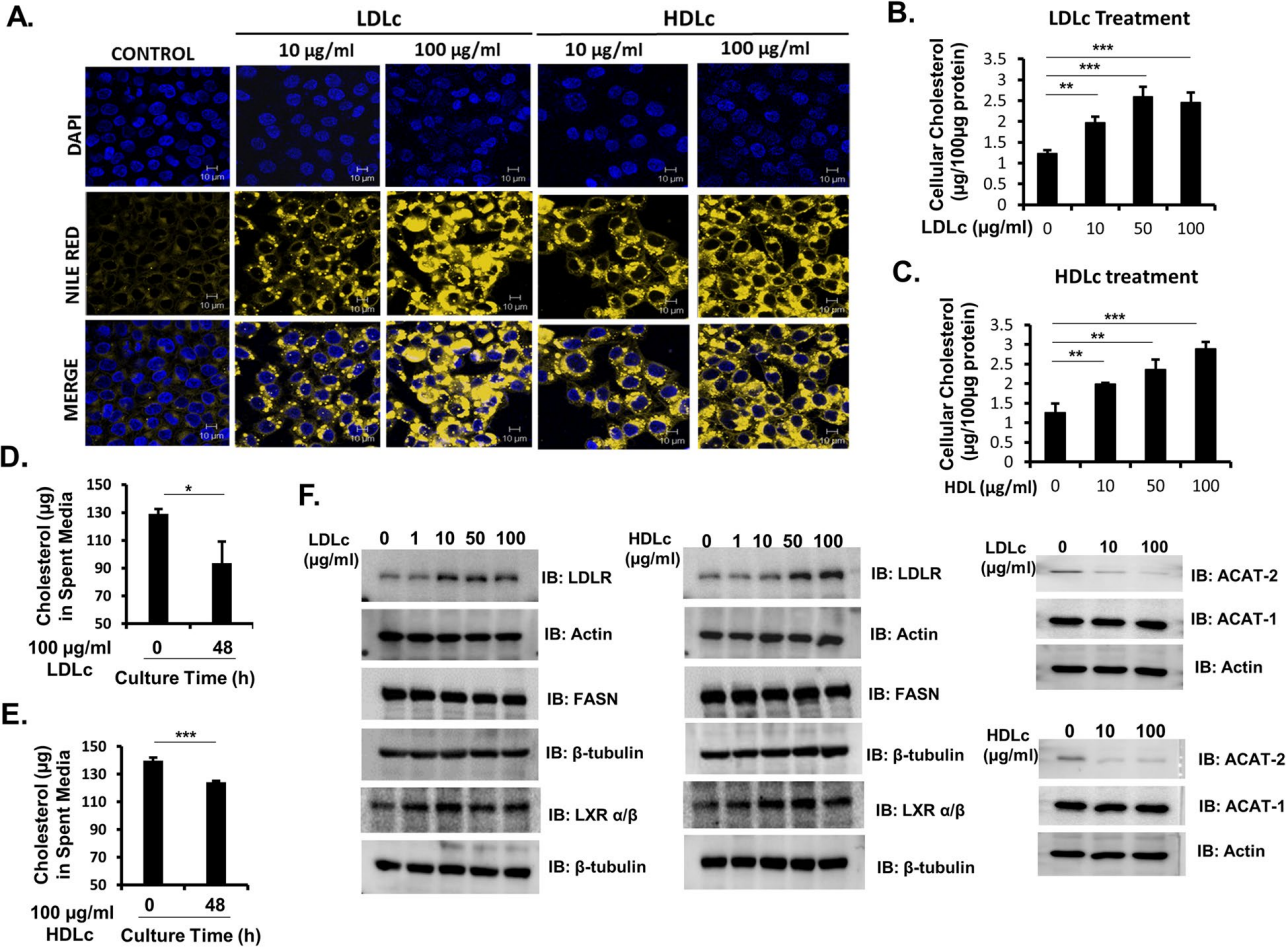
Cellular and molecular changes in the lipid metabolism upon LDLc and HDLc treatment. Cells were treated with the desired concentration of LDLc/HDLc/vehicle as shown in the figure, for 48 h followed by the analysis of molecules involved in lipid metabolism through immunoblotting, and quantification of lipid accumulation (Nile red staining) through confocal, or cholesterol accumulation analysis through cholesterol estimation kit. **A** Confocal images of HCT-116 cells treated with or without LDLc/HDLc after staining with Nile red. Experiments were done in triplicate and performed twice. **B**,**C** Estimation of intracellular cholesterol from the whole cell lysate of HCT-116 cells treated with or without LDLc/HDLc. The experiment was done in triplicate. **D**,**E** Cholesterol estimation from the spent media of HCT-116 cells after treatment with or without LDLc/HDLc. The experiment was done in triplicate. **F** Immunoblot analysis of molecules involved in lipid metabolism of colon cancer cells after LDLc or HDLc treatment. HCT-116 cells were exposed to different concentrations of LDLc or HDLc as shown in the figure, and immunoblotting for LDLR, FASN, LXRα/β, ACAT-1, and ACAT-2 were performed in the whole cell lysate. Actin and β-tubulin were used as the loading control

Cancer cells require an excessive amount of nutrients including cholesterol for which they rely either on rapid uptake of cholesterol or upregulation of cholesterol biosynthesis [31, 32]. Firstly, the amount of intracellular lipid (detected by Nile red staining) and cholesterol accumulation in HCT-116 and HCT-15 cells were examined in the presence or absence of extracellular LDLc or HDLc. The amount of intracellular lipid accumulation in HCT-116 was significantly higher in the cells treated with LDLc or HDLc as compared to their respective untreated/vehicle controls, in a time and concentration-dependent manner (Fig. 4A–C; Supplementary Fig. 2). A similar increase in lipid accumulation was also observed in HCT-15 cells (Supplementary Fig. 3). Interestingly, a difference in the rate of lipid accumulation between LDLc-treated and HDLc-treated cells was observed. Rapid accumulation of lipids was detected in HDLc as compared to LDLc treatment (Supplementary Fig. 2A-C). Furthermore, intracellular cholesterol accumulation was also increased by more than twofold in HCT-116 cells supplemented with LDLc or HDLc as compared to the untreated control (Fig. 4B,C). Parallelly, the amount of cholesterol used up by the HCT-116 cells after treatment with vehicle or LDLc or HDLc was also analyzed. A significant decrease in the amount of cholesterol in the spent media of HCT-116 cells treated with LDLc or HDLc was detected in comparisons to their respective controls’ indicative of their utilization by the cells from the culture medium (Fig. 4D,E). From this experiment, it is evident that t colon cancer cells can uptake cholesterol (LDLc or HDLc) from the extracellular environment which can be stored as lipid droplets inside the cells. The presented experimental results are also in agreement with the finding by Wang et al., in which they have shown that the level of cholesterol accumulation was increased in isolated intestinal crypt upon supplementation with cholesterol [10]. Somatic mutations have been previously linked to upregulated de novo cholesterol biosynthesis in different colon cancer types [33]. Therefore, the role of various somatic mutations, such as APC/KRAS in regulating cholesterol uptake or lipid accumulation cannot be ignored.

Because a substantial increase in the accumulation of lipid/cholesterol in HCT-116 cells was observed, we further evaluated the effect of extracellular LDLc or HDLc on the expression of molecules associated with cholesterol uptake or esterification.

Many cancers including colon cancer show an upregulated LDLR expression [31, 32]. Based on TMN plotter analysis performed on the unpaired and paired samples of normal, tumor, and metastatic colon tissue. We observed an increase in gene expression of LDLR in colon tumor and metastatic tissue compared to normal counterparts (Supplementary Fig. 7.2 A). Studies in breast cancer have also correlated the expression of LDLR with the increase in tumor progression and cancer cell proliferation in settings with elevated LDLc levels [34]. The expression level of LDLR in HCT-116 cells in the presence or absence of LDLc or HDLc was evaluated. Immunoblot analysis shows an increase in the level of LDLR in both LDLc- and HDLc-treated conditions as compared to vehicle control (Fig. 4F), suggestive of its role in rapid cholesterol uptake. Similarly, increase in the protein expression of LDLR in the tumor tissue lysates from HFD and HCD mice iso-grafted with MC-38 cells was detected as compared to the tumor lysates from ND control mice (Supplementary Fig. 9.B). Furthermore, to check the status of lipid metabolizing enzymes, the expression levels of fatty acid synthase (FASN), Acyl-coenzyme A cholesterol acyl transferases 1 (ACAT1), and Acyl-coenzyme A cholesterol acyltransferases 2 (ACAT2) were examined. FASN is an important multi-enzyme protein that regulates fatty acid biosynthesis (de novo lipogenesis) in cancer cells and provides fatty acids as a source of energy to the proliferating cells. Similarly, ACATs perform the function of cholesterol esterification. Immunoblot analysis in HCT-116 cells shows a decrease in the protein level of FASN and ACAT-2. However, no noticeable difference in the protein level of ACAT-1 was observed (Fig. 4F). Moreover, the level of sterol sensing receptor LXR (Lipid X Receptor) and the scavenger receptor, Class B1 (SR-B1/SCARB-1) which is considered as a receptor for HDLc were also evaluated in HCT-116 cells. Treatment with LDLc or HDLc shows an increase in the expression of LXR α/β whereas no significant alteration of SCARB-1 was observed upon LDLc treatment but the expression of SCARB-1 was upregulated upon HDLc treatment in HCT-116 cells (Fig. 4F; Supplementary Fig. 9A).

Even though a direct correlation between lipid/cholesterol accumulation in cancer cell proliferation is unclear, it is likely that the changes in lipid metabolism associated with LDLc or HDLc may support proliferation. Collectively, supplementation of LDLc or HDLc alters the lipid metabolism of colon cancer cells by increasing cholesterol accumulation and lipid droplet formation along with alteration in the expression of molecules involved in lipid metabolism.

### LDLc and HDLc enhance glucose uptake, utilization, and lactate secretion

**Fig. 5:**
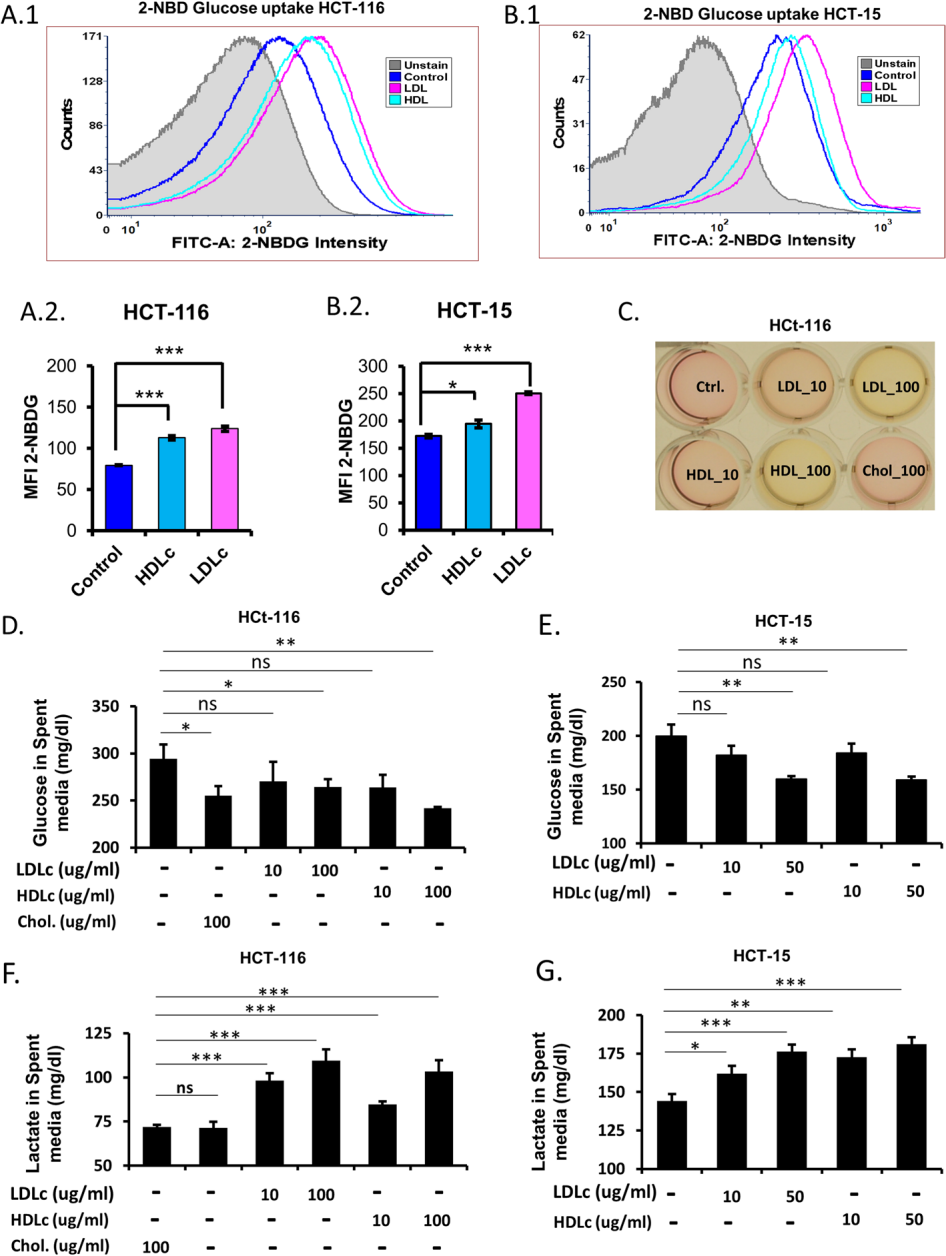
LDLc and HDLc regulate glucose uptake, glucose utilization, and lactate secretion in colon cancer cells. **A**, **B** 2-NBD Glucose uptake assay in colon cancer cells. HCT-116 and HCT-15 cells were exposed to LDLc (50 µg/ml) and HDLc (50 µg/ml) or vehicle for 24 h and subjected to 2-NBD Glucose treatment with or without LDLc/HDLc (50 µg/ml) in glucose-free media for 30 min and analyzed through BD FACS CANTO II. **A**.**1**, **A**.**2** Histograms of 2-NBDG in different treated conditions in HCT-116 and their respective MFIs. **B**.**1**, **B**.**2** Histograms of 2-NBDG in different treated conditions in HCT-15 and their respective MFIs. Experiments were done in triplicate and performed twice. **C–G** Glucose and lactate estimation in the spent media of LDLc or HDLc or vehicle-treated HCT-116 and HCT-15 cells. For glucose utilization and lactate production analysis, glucose, and lactate present in spent media were quantified after 24 h treatment with different concentrations of LDLc or HDLc or free cholesterol or vehicle as described in the method section. **C** Image of spent media from HCT-116 cells cultured in different treatment conditions. **D**, **F** Lactate and glucose estimation in spent media of HCT-116 cells. **E**,**G** Estimation of lactate and glucose in the spent media of HCT-15 cells. Experiments were done twice in triplicate

Aberrant glucose metabolism is an important contributor to the metabolic reprogramming of cancer cells and is regarded as an emerging hallmark of cancer [21]. Cancer cell growth and proliferation are often linked with the availability of nutrients, i.e., in particular glucose, as a main source of energy. Interestingly, supplementation of LDLc or HDLc in the culture medium of HCT-116 cells leads to rapid change in the media color as compared to untreated control, which is indicative of a change in the pH of media due to lactate accumulation (acidification) (Fig. 5C). Based on these observations, we speculated that glucose metabolism is deregulated in cells treated with LDLc or HDLc. Various parameters associated with glucose metabolism, i.e., glucose uptake, glucose utilization, lactate secretion, were analyzed in cells treated with cholesterol. First, the effect of LDLc and HDLc on glucose uptake was analyzed by using fluorescently labelled glucose, i.e., 2-NBDG through FACS. A significant increase in the uptake of 2-NBDG by HCT-116 (Fig. 5A1,A2) and HCT-15 (Fig. 5B1,B2) cells was observed when supplemented with LDLc or HDLc. As the presence of LDLc or HDLc increases 2-NBDG uptake, its effect on the utilization of glucose was also checked. Treatment of LDLc or HDLc in HCT-116 and HCT-15 cells promotes glucose utilization in a dose-dependent manner as compared to untreated control (Fig. 5D,E). The increase in glucose uptake and utilization is likely to affect lactate production, a by-product of aerobic glycolysis which is reported to impact migration, metastasis, survival, radioresistance, chemoresistance, etc. [25, 35]. Therefore, the amount of lactate secreted by cells in the spent media was estimated with or without LDLc or HDLc treatment. Supplementation of LDLc or HDLc in the culture medium of HCT-116 and HCT-15 cells enhance lactate secretion in a dose-dependent manner (Fig. 5F,G). Collectively, an increase in glucose uptake, its utilization, and lactate secretion upon LDLc or HDLc treatment is a consequence of alteration in glucose metabolism in these cells which might play a dynamic role in promoting the growth and proliferation of cells.

### LDLc and HDLc activates glycolytic enzyme and upregulates the expression of lactate transporter

**Fig. 6:**
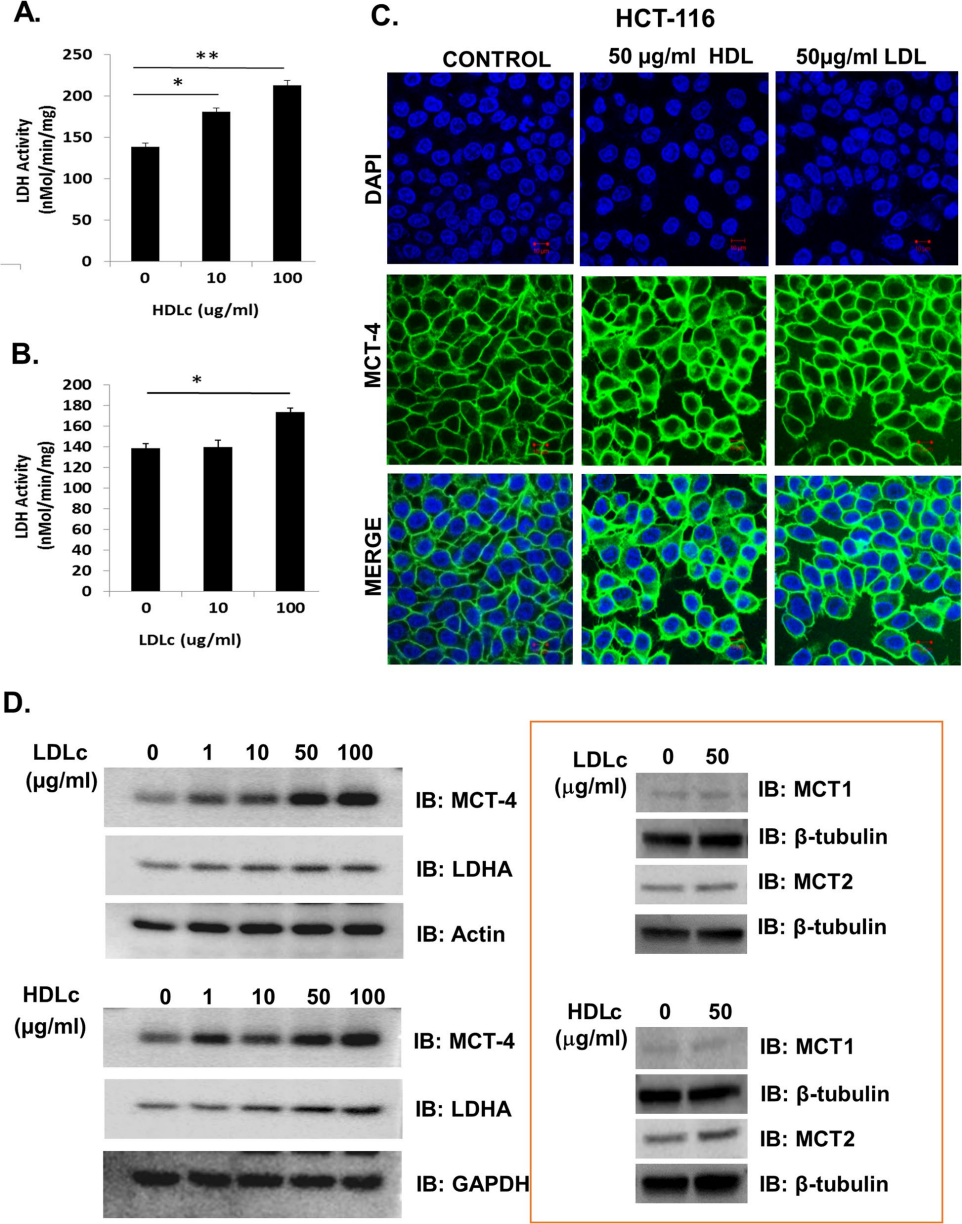
LDLc and HDLc interfere with enzymes and molecules involved in glucose metabolism, i.e., lactate production and transporter in colon cancer cells. Cells were treated with different concentrations of LDLc or HDLc or vehicle and enzyme activity assay, immunofluorescence, and western blotting were performed. **A**,**B** Lactate dehydrogenase enzyme activities upon **A** HDLc and **B** LDLc treatment in HCT-116 cells. **C** Confocal images of HCT-116 cells probed with MCT-4. Experiments were done in triplicate. **D** Immunoblot analysis of MCT-1, MCT-2, MCT-4, and LDHA in HCT-116 whole cell lysate. β-tubulin, Actin, and GAPDH were used as loading controls. The experiment was performed twice

To further investigate the role of LDLc and HDLc in glucose metabolism, the status of lactate dehydrogenase (LDH) activity (vital enzyme for lactate production) was analyzed, as this enzyme has previously been linked to tumorigenesis [36]. Also, in vitro treatment of HCT-116 cells with LDLc or HDLc causes increases in the expression of LDHA and its enzymatic activity in comparison with the untreated control (Fig. 6A, D). Additionally, an increase in LDHA protein level was detected by immunoblot analysis in the lysates of MC-38 isografted tumor tissue from HCD and HFD mice as compared to ND control mice (Supplementary Fig. 9B).

Lactate transport across the cell membrane is facilitated by specific transporters and Monocarboxylate transporter 4 (MCT-4) is a major lactate transporter that is frequently overexpressed in numerous cancers. Previous studies have shown the significance of MCT-4 in the proliferation of various cancer cells including colon cancer [37]. Immunoblotting and immunofluorescence analysis of HCT-116 cells treated with LDLc or HDLc show an increase in the protein level of MCT-4 as well as its localization on the membrane in comparison to untreated control (Fig. 6C,D). In HCT-15 cells also, MCT-4 level was increased (Supplementary Fig. 4). However, the protein levels of other lactate transporters (MCT-1 or MCT-2) were not significantly altered in HCT-116 and HCT-15 cells treated with LDLc or HDLc, whereas an increase in MCT-3 was observed in HCT-15 cells only (Supplementary Fig. 4). An increase in the protein level of MCT-4 by western blotting was also detected in the MC-38 iso-grafted tumor tissue lysate from HFD mice but no significant difference was observed in HCD as compared to ND mice (Supplementary Fig. 9B). Collectively, these results indicate that, in the presence of LDLc or HDLc, lactate transport is primarily facilitated by MCT-4. Taken together, in the presence of LDLc or HDLc, colon cancer cells reprogram their cellular machinery associated with glucose metabolism for sustaining rapid proliferation.

### LDLc or HDLc potentiates aerobic glycolysis

**Fig. 7:**
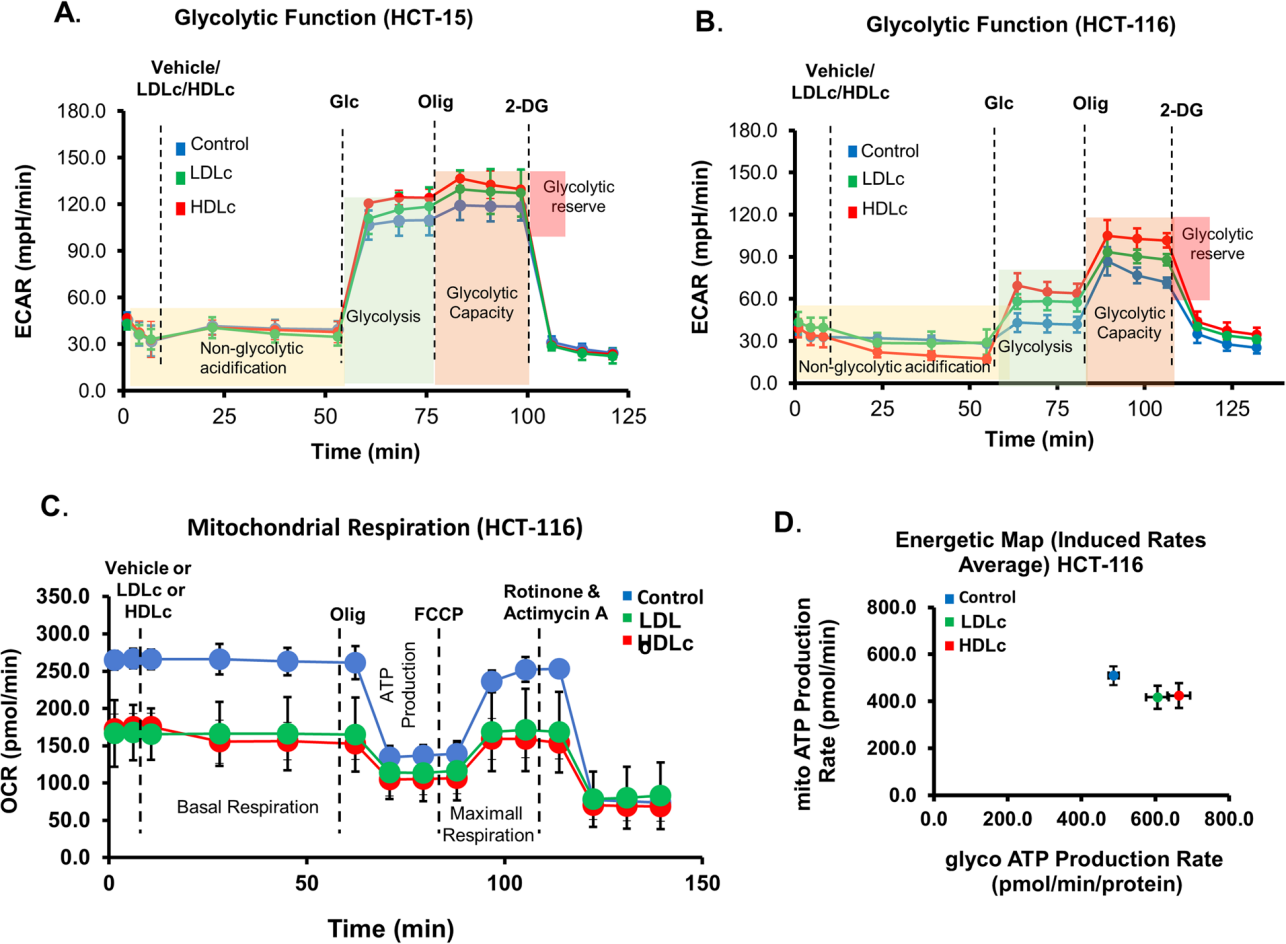
LDLc and HDLc regulate glycolytic function, mitochondrial respiration, and ATP production in colon cancer cells. HCT-116/ HCT-15 cells were pre-treated with 50 μg/ml LDLc/HDLc for 12 h in 1% FBS-containing media followed by the analysis of various metabolic parameters in the presence of 50 μg/ml LDLc/HDLc through XeF24 seahorse analyzer. **A**,**B** Glycolytic stress (ECAR) functional analysis in HCT-15 and HCT-116 respectively. Experiments were performed twice in triplicate (except for the HCT-116 control condition, done in duplicate). **C** Graph showing mitochondrial stress (OCR) functional analysis in HCT-116 cells. Experiments were performed twice in triplicate. **D** Seahorse XeF real-time ATP rate analysis of HCT-116 cells upon the vehicle or LDLc or HDLc treatment. The experiment was done in triplicate

The significance of LDLc and HDLc in the glucose metabolism of normal cells has been reported [17–19]. Since the presence of LDLc or HDLc increases glucose uptake, utilization, and lactate secretion in HCT-116 and HCT-15 cells, we further probed into the real-time effect of LDLc or HDLc on the aerobic glycolysis, and ATP production in cells using Seahorse XFe24 analyzer (Agilent Technologies, Inc., USA). Extracellular acidification rate (ECAR) was increased in HCT-116 and HCT-15 cells treated with LDLc or HDLc, compared to untreated control (Fig. 7A,B; Supplementary Fig. 5A, B). The increase in ECAR ascertains the upregulation of glucose metabolism through enhanced glycolytic processes (i.e., increase in glycolysis and glycolytic reserve).

As upregulated glycolysis is reflected by the increase in total ATP production through aerobic glycolysis. Therefore, the amount of total ATP generated from aerobic glycolysis and OXPHOS in HCT-116 cells was evaluated in the presence or absence of LDLc or HDLc. A significant increase in the level of ATP generated through aerobic glycolysis was observed in LDLc- or HDLc-treated cells as compared to untreated/vehicle control, with an approximation of 12 and 9.3% increase in glycolytic ATP generation upon HDLc and LDLc treatment, respectively (Fig. 7D; Supplementary Fig. 5D). Parallelly, a concomitant decrease in the amount of OXPHOS-mediated ATP generation by about 12% in HDLc- and 9% in LDLc-treated HCT-116 cells was observed when compared to untreated control (Fig. 7D; Supplementary Fig. 5D). These results ascertain the role of LDLc or HDLc in enhancing the glycolytic process and ATP generation in cancer cells.

### LDLc or HDLc treatment decreases mitochondrial respiration and mitochondrial density

As there is an increase in glycolytic rate and a change in ATP generation through OXPHOS as well as aerobic glycolysis in HCT-116 and HCT-15 cells in the presence of LDLc or HDLc, the effect of cholesterol on the real-time analysis of mitochondrial respiration [oxidative phosphorylation (OXPHOS)] in the HCT-116 cells was evaluated. A significant decrease in the level of oxygen consumption rate (OCR) was recorded in LDLc- or HDLc-treated conditions compared to untreated control (Fig. 7C; Supplementary Fig. 5C), indicative of a reduction in the mitochondrial respiration through OXPHOS in these cells. Also, a substantial decrease in basal respiration, maximal respiration, and proton leak was prominent in LDLc- or HDLc-treated conditions. In addition, by the mitostress assay, a decrease in ATP production was observed in the treated conditions when compared to the control (Fig. 7C; Supplementary Fig. 5C), which is in accordance with the earlier real-time ATP rate analysis.

As the overall mitochondrial respiration rate was decreased, the effect of LDLc or HDLc on the mitochondrial density of HCT-116 and HCT-15 cells was further evaluated using Mito tracker dye. Treatment with LDLc or HDLc decreases the mitochondrial density in both cells (Supplementary Fig. 6A-D), which is likely to contribute to diminished OXPHOS activity.

Collectively, the abovementioned results indicate that treatment of cells with LDLc or HDLc interferes with glucose metabolism by promoting aerobic glycolysis and decreasing mitochondrial respiration, thus causing a metabolic shift towards a more glycolytic phenotype.

### Oxamate abrogates the proliferative action of LDLc and HDLc by inhibiting aerobic glycolysis and promoting cell death

**Fig. 8:**
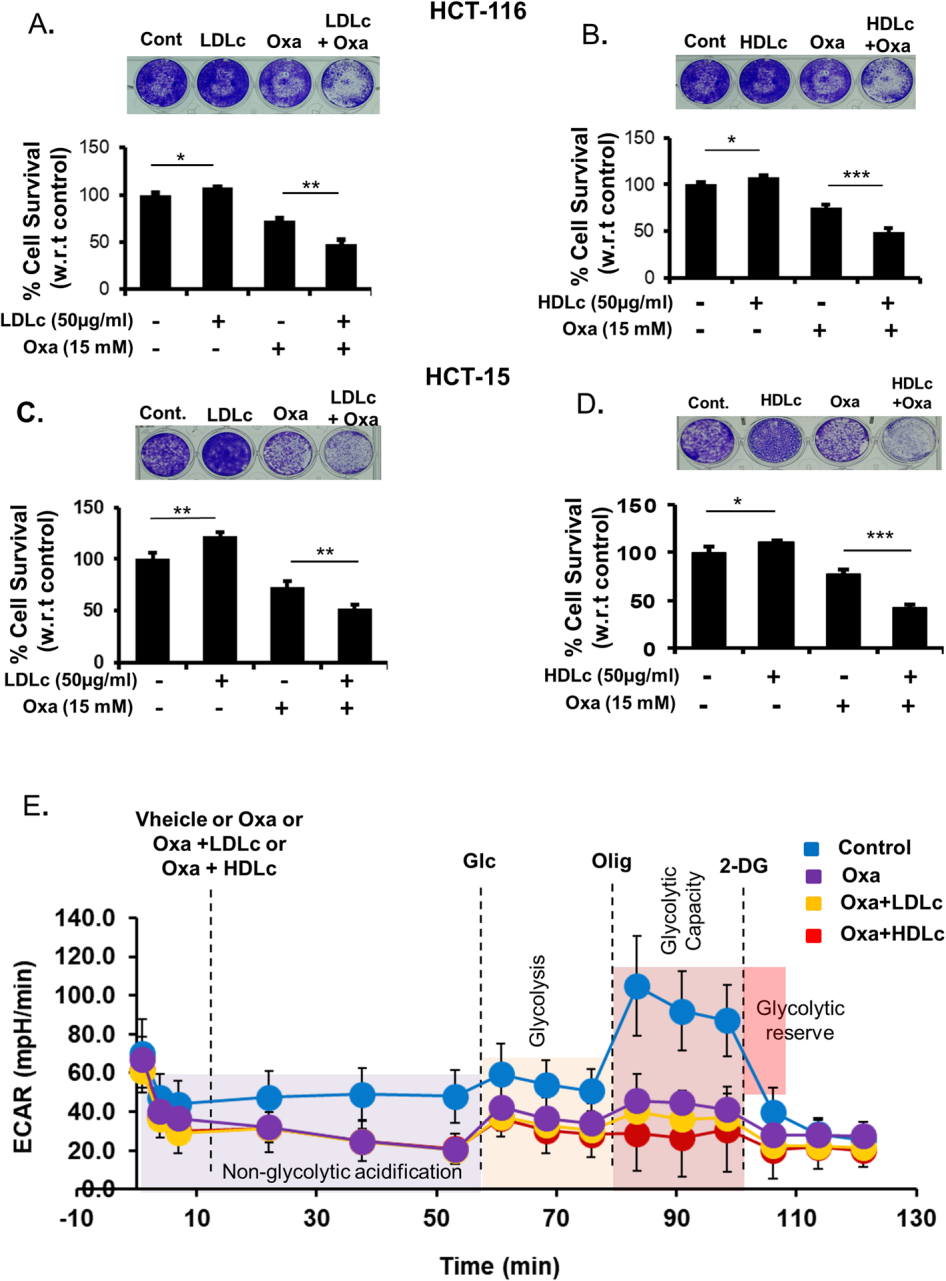
Oxamate abrogates LDLc or HDLc promoted proliferation of colon cancer cells. **A-D** Crystal violet staining for quantification of cell survival/proliferation. HCT-116 and HCT-15 cells were treated with oxamate (15 mM) in the presence or absence of LDLc/HDLc (50 μg/ml) for 48 h, and crystal violet staining was performed. **A**, **B** HCT-116 treated with oxamate in the presence or absence of LDLc and HDLc respectively. **C**,**D** HCT-15 cells treated with oxamate in the presence or absence of LDLc or HDLc respectively. Bar graphs represent the relative intensity indicative of % survival. Experiments were done in triplicate. **E** Targeting the glycolytic function of colon cancer cells with oxamate treatment in the presence or absence of LDLc or HDLc**.** HCT-116 cells were pre-treated with vehicle or 50 μg/ml of LDLc/HDLc with or without oxamate for 12 h in 1% FBS-containing media, followed by the analysis of various glycolytic parameters in the presence of vehicle or 50 μg/ml LDLc/HDLc through XeF24 seahorse analyzer. The graph represents the glycolytic stress (ECAR) functional analysis. The experiment was done in triplicate

To abrogate the enhancement in glycolysis triggered by LDLc and HDLc, an inhibitor of glycolysis, i.e., oxamate (a competitive inhibitor of LDH enzymes), was utilized. Earlier studies have reported the significance of targeting glucose metabolism for controlling cancer cell growth and proliferation. Oxamate is one such inhibitor that is reported to exhibit promising anticancer properties either alone or in combination with other drugs [38, 39]. Therefore, the effect of oxamate was evaluated in HCT-116 and HCT-15 cells in the presence or absence of LDLc or HDLc (oxamate-LDLc/HDLc) by assaying cell survival through violet staining assay. A significant decrease in cell survival was observed in HCT-116 and HCT-15 cells when treated with oxamate in the presence of LDLc/HDL conditions as compared to oxamate alone or vehicle controls (Fig. 8A–D).

Also, oxamate treatment causes significant inhibition in aerobic glycolysis induced by LDLc or HDLc supplementation (Fig. 8E). Therefore, as is evident from the mitostress assay result, LDLc or HDLc treatment decreases mitochondrial respiration and causes a shift in the metabolism towards a more glycolytic phenotype. Subsequently, the presence of oxamate blocks the glycolytic pathway thereby exhibiting an additive effect in reducing the proliferation of colon cancer cells (combination treatment condition). Taken together, these experimental results indicate that inhibition of LDH enzymes by oxamate nullifies the proliferative action of LDLc or HDLc and inhibits the proliferation of cells.

### Inhibition of glucose metabolism by 2-DG does not prevent lipid accumulation

We also looked into the effect of targeting glucose metabolism in the context of lipid droplet accumulation in the cells. 2-DG is a glucose analog that inhibits glycolysis and binds with the hexokinase enzyme of the glycolytic pathway thereby impeding the glucose metabolism. Therefore, an experiment was performed to evaluate whether inhibition of glucose uptake by 2-DG has any consequence on lipid/cholesterol accumulation in the cells treated with LDLc or HDLc. An increase in lipid accumulation was detected by Nile red staining of the cells exposed to LDLc or HDLc treatment in the presence of 2-DG (Supplementary Fig. 8). This result indicates that abrogation of glucose metabolism alone does not prevent lipid accumulation in colon cancer cells even though it causes a reduction in cell proliferation.

### Clinical significance and the correlation of the molecules involved in lipid and glucose metabolism in colon cancer prognosis

Our in vitro and in vivo findings are indicative of a significant correlation between altered lipid and glucose metabolism in colon cancer cells upon cholesterol availability. Subsequently, we performed the Spearman correlation analysis of two important gene, i.e., LDLR and LDHA involved in lipid uptake and glucose metabolism from colon cancer patient data using TMN plotter (Supplementary Fig. 7.1). A positive correlation in the gene expression profile of LDLR and LDHA was observed in cancer patients, suggestive of the involvement of these two genes in colon cancer tumorigenesis. Furthermore, gene expression profile of LDLR, LDHA, FASN, and MCT-4 were also performed in normal, tumor, and metastatic human colon tissue through TNM plotter. TMN plotter analysis of unpaired and paired data sets show a significant increase in the gene expression profile of LDLR, LDHA, and FASN in tumor and metastatic tissues as compared to normal colon tissue. However, no significant difference was observed in the expression of MCT-4 between normal and cancer tissues (Supplementary Fig. 7.2A-D). To further provide the clinical relevance of our findings, Kaplan–Meier curves for colon cancer patient survival were generated by Kaplan–Meier plotter (KM Plotter). Kaplan–Meier survival curves were plotted with respect to the expression profile of the gene of interest involved in lipid (LDLR & FASN) and glucose metabolism (LDHA & MCT-4). A detailed analysis performed by KM plotter clearly indicates that overexpression of LDLR correlates with poor prognosis in colon cancer patients regardless of the tumor stage and gender (Supplementary Fig. 7.3–7.5). Furthermore, survival analysis was also performed in the context of high LDLR with high and low LDHA or MCT-4 or FASN expression. A significant decrease in the survival was evident in all the colon cancer patients having higher LDLR with higher LDHA or MCT-4 or FASN expression in comparison to higher LDLR with lower LDHA or MCT-4 or FASN expression (Supplementary Fig. 7.6—7.8). Statistical significance of all the survival analysis data is given in Supplementary tables 2 and 3.

Collectively, probing into colon tissue gene expression databases indicates upregulation of LDLR, FASN, LDHA, and MCT-4 in tumor tissues, which are in line with our preclinical findings. Furthermore, analysis of colon cancer patient survival databases points towards correlation between upregulation of these molecules with poor clinical outcome.

## Discussion

Malignant cells have the ability to hijack cholesterol metabolism to support tumorigenesis, stemness, survival, and metastasis. Previous clinical studies in colon cancer patients have reported an alteration in the blood cholesterol level during the progression of the disease [16]. However, the impact of enhanced dietary cholesterol intake and increased blood cholesterol levels in colon cancer initiation and progression is least explored at the molecular level and still elusive. Additionally, the mechanism by which cholesterol regulates cancer cell proliferation is still not clearly understood. Few studies do suggest the involvement of cholesterol in various signaling pathways as an intermediate to support the survival and proliferation of cancer cells.

To provide insights into missing links between the cholesterol-colon cancer correlation, the hypercholesterolemic mice model was developed in C57BL/6 J mice through supplementation of high-cholesterol or high-fat diet, and its effect on colon cancer initiation and progression was evaluated. An increase in the incidence of polyps’ formation and rapid growth of cancer cell isografted tumors in mice supplemented with HCD or HFD compared to ND indicates the involvement of cholesterol in supporting colon cancer initiation and progression. As evident from the earlier reports, dyslipidemia because of obesity or high-cholesterol diet can disrupt the intestinal protective layers because of high inflammatory state leading to an imbalance in the recovery and damage of intestinal tissue [40]. This might also play a significant role in the development of the AOM/DSS-induced colon cancer model. Collectively, cholesterol either in the blood or in diet may have a positive influence on these colonic cells during the process of tumorigenesis. Moreover, the effect of cholesterol on proliferation was also evaluated in various colon cancer cells upon supplementation with LDLc or HDLc, in culture conditions. The availability of extracellular cholesterol facilitates cells to proliferate. Through animal-based and cell lines studies, supplementation of cholesterol either in culture conditions or in the diet has a profound impact on colon cancer cell proliferation, migration, tumor initiation, and progression, which are in agreement with the earlier findings in various cancers [10, 14, 15].

Enhanced proliferation and aggressiveness of the tumor are often correlated with alteration in metabolic parameters. Starting from the onset of cancer till the disease progresses through various stages, two important metabolic parameters are frequently deregulated, i.e., alteration in lipid as well as glucose metabolism. Majorities of cancer cells exhibit alterations in the cellular lipid metabolism either by increasing lipid biogenesis or through enhanced lipid uptake by overexpressing genes and proteins involved in lipid biogenesis or uptake (such as SREBP, LXR, FASN, and LDLR) [16]. Overexpression of LDLR in the tumor tissue of colon and its correlation with the decrease in the survival of colon cancer patients is indicative of the involvement of altered lipid metabolism in increasing the severity of colon cancer tumorigenesis. However, several external factors or stimulus through which these alterations in metabolism are being regulated is yet to be explored in most cancers. Therefore, various in vitro experiments were designed for probing into the underlying molecular mechanism through which cholesterol regulates cancer cell proliferation and its correlation with cellular metabolism. The effects of extracellular cholesterol (LDLc and HDLc) on the glucose and lipid metabolism of colon cancer cells were evaluated. Interestingly, we found a significant increase in the amount of lipid or cholesterol accumulation inside the cells along with upregulated LDLR and downregulated FASN protein expression (a master regulator of lipid metabolism) upon cholesterol availability. The decrease in FASN with the availability of cholesterol or lipid is suggestive of these cells relying more on exogenous cholesterol/lipid uptake which is a less energy-consuming process instead of more energy-consuming endogenous biosynthesis. It is well documented that lipid droplet accumulation performs multiple functions in regulating hallmarks of cancer, including modulation of signaling pathways, cell cycle regulation, tumor and immune cell crosstalk, hypoxia-mediated alteration in lipid metabolism [41]. Moreover, previous studies suggest that lipid droplets can also behave like an organelle and are involved in various important functions such as storage of lipids, regulation of lipid metabolism, membrane trafficking, and as a mediator of various inflammatory signaling molecules [42, 43]. Available literature along with our experimental findings is suggestive of the role of LDLc and HDLc in tuning the lipid metabolism to support the rapid proliferation of cells.

Additionally, we further looked into the role of cholesterol in the glucose metabolism of colon cancer cells. Previous studies have reported an interrelationship between altered blood cholesterol (LDL or HDL) levels with the risk of type 2 diabetes (T2D) [17, 44]. Familial hypercholesterolemic patients were reported to have a decreased risk of type 2 diabetes. In addition, a decrease in the level of LDLc in blood, through lipid-lowering agents decreases the risk of cardiovascular diseases but increases the risk of T2D [44]. Moreover**,** HDLc has also been shown to regulate blood glucose levels through enhanced glucose uptake (insulin-independent) by skeletal muscle [17]. Likewise, enhanced glucose uptake was also observed in HDLc-treated adipocytes [18].

Additionally, correlation analysis of LDLR and LDHA gene expression profile shows a positive association of these two gene in colon cancer patients. As evident from various clinical data, the decrease in the survival of colon cancer patients was highly correlated with the overexpression of molecules involved in cholesterol uptake (i.e., LDLR) and glucose metabolism, i.e., LDHA or MCT-4. Upregulated expression of both LDLR with LDHA or MCT-4 significantly worsen the survival of colon cancer patients.

Collectively, these studies point towards the important role of cholesterol in influencing cellular glucose metabolism of normal cells. However, no such reports are available in the context of cancers, though alteration in glucose metabolism is considered as an emerging hallmark [21]. Numerous cancer types rely more on aerobic glycolysis than OXPHOS for ATP generation [45]. A recent study from Broadfield et al. has reported the role of fat in supporting the initiation of hepatocellular carcinoma through the upregulation of glucose metabolism [20]. Furthermore, the present study puts forth important factors (i.e., LDLc or HDLc) which can influence the glucose metabolism of colon cancer cells thereby supporting proliferation. The ability of colon cancer cells to increase glucose metabolism by upregulating aerobic glycolysis in the presence of LDLc or HDLc is advantageous for their rapid cell proliferation. The availability of LDLc or HDLc in the microenvironment facilitates cells in enhancing the glycolytic process leading to increased glucose uptake, utilization, and lactate production. Shifting ATP production towards a more glycolytic phenotype from the conventional OXPHOS-mediated ATP synthesis (which is considered to be a slower process) helps in rapid ATP synthesis. These changes in the metabolic processes upon LDLc or HDLc availability might support the increasing need for energy (ATP) for the proliferation of cancer cells. Moreover, various molecules involved in the cellular glucose and lipid metabolism such as LDLR, LDHA, and MCT-4, in this study, are also of significant clinical relevance as they were shown to be directly linked with colon cancer patient survival. Collectively, our investigation highlights an important distinctive feature of colon cancer cells by which their cellular metabolism is reprogrammed in the presence of cholesterol to support rapid growth.

We also put forth the relevance of targeting glucose metabolism towards controlling colon cancer cell proliferation in a cholesterol-rich environment. Previous studies also emphasized inhibiting glucose metabolism by targeting LDH enzyme as a potential target for various cancer [2, 46]. Herein, oxamate, an inhibitor of LDH enzyme for targeting cholesterol-induced upregulated glycolysis, was utilized. It is likely that the ability of LDLc and HDLc to cause a decrease in OXPHOS along with a reduction in mitochondrial density may contribute to the enhancement of oxamate-induced cell death through inhibition of aerobic glycolysis. As the population of hypercholesterolemic and obese individuals is on an upward trend globally, therefore the utility of glycolytic inhibitors such as oxamate may be of relevance in a subset of colon cancer patients with high blood cholesterol.

## Conclusion

In conclusion, this study highlights the functionality of increased cholesterol availability through diet or obesity in promoting colon cancer cell proliferation, tumor initiation, and tumor progression. Colon cancer cells have the ability to utilize both LDLc as well as HDLc, leading to alteration in cellular metabolism of glucose (i.e., shifting towards a more glycolytic and aggressive phenotype) and lipids, thereby supporting the rapid proliferation of cells. Additionally, targeting the aberrant glucose metabolism through LDHA inhibitors can restrict cholesterol-induced colon cancer cell proliferation. This study opens avenues for investigating the interrelationship between high blood cholesterol levels with the etiology of various cancers and also has implication in therapeutic interventions for cancers.

### Supplementary Information

**Additional file 1:** **Supplementary figure 1. **Effect of LDLc and HDLc supplementation on the proliferation of colon cancer cells.  **Supplementary figure 2. **Time-dependent lipid accumulation by colon cancer cells upon LDLc or HDLc treatment. **Supplementary figure 3. **Lipid accumulation upon LDLc and HDLc treatment in HCT-15 cells. **Supplementary figure 4. **Immunoblot analysis of molecules associated with colorectal cancer cells proliferation after LDLc or HDLc treatment. **Supplementary figure 5. **Role of LDLc and HDLc in glycolytic function, mitochondrial respiration, and ATP production in colon cancer cells. **Supplementary figure 6. **Role of LDLc and HDLc in the mitochondrial biogenesis in colon cancer cells. **Supplementary figure 7.1**. Correlation between LDLR and LDHA gene expression in colon cancer patients. Data was extracted using TMNplotter. **Supplementary figure 7.2.** Gene expression profile of LDLR, LDHA, MCT-4, and FASN in tumor, non-tumor and metastatic tissue of human colon from Gene CHIP Data.  **Supplementary figure 7.3.** Kaplan-Meier curves for survival of colon cancer patients by LDLR, FASN, LDHA, and MCT4 status. **Supplementary figure 7.4.** Kaplan-Meier curves for Overall survival, Relapse free survival, and Post progression survival of colon cancer patients in male and female by high and low LDLR expression. **Supplementary figure 7.5.** Kaplan-Meier curves for Overall survival, Relapse free survival, and Post progression survival of colon cancer patients in different stages (I-IV) by high and low LDLR expression. **Supplementary figure 7.6.** Kaplan-Meier curves for survival of colon cancer patients with high LDLR expression together with  high or low LDHA, FASN, and MCT-4 expression. **Supplementary figure 7.7.** Kaplan-Meier curves for Overall survival, Relapse free survival, and Post progression survival of colon cancer patients in male and female by high LDLR with low LDHA expression in comparison to high LDLR with high LDHA expression. **Supplementary figure 7.8.** Kaplan-Meier curves for Overall survival, Relapse free survival, and Post progression survival of colon cancer patients in different stages (I-IV) by High LDLR with low LDHA expression in comparison to High LDLR with high LDHA expression. **Supplementary figure 8. **Effect of 2-DG (2 Deoxy-D-glucose) on lipid accumulation in cells treated with LDLc and HDLc. **Supplementary figure 9. **Immunoblot analysis in lysates of HCT-116 cells and MC-38 tumor. **Supplementary table 1. **Serum parameters changes in ND, HFD, and HCD pooled mice serum. **Supplementary table 2. **High expression of LDLR results in poor prognosis of colon cancer patient. **Supplementary table 3. **Survival data of colon cancer patients expressing high LDLR with low or high LDHA expression.

**Additional file 2.**

## Data Availability

Not applicable. However, raw immunoblots used in this manuscript have been submitted.

## Abbreviations

LDLc: Low-density lipoprotein cholesterol
HDLc: High-density lipoprotein cholesterol
ATP: Adenosine triphosphate
OXPHOS: Oxidative phosphorylation
ISC: Intestinal stem cells
DMEM: Dulbecco’s modified Eagle’s medium
FBS: Fetal bovine serum
DSS: Dextran sodium sulfate
AOM: Azoxymethane
EAF: Experimental Animal Facility
ND/HFD/HCD: Normal diet/ High-fat diet/ High-cholesterol diet
PBS: Phosphate buffer saline
CPCSEA: Committee for the Purpose of Control and Supervision of Experiments on Animals
IAEC: Institutional Animal Ethics Committee
PFA: Paraformaldehyde
DAPI: 4,6-Diamidino-2-phenylindole
2-NBDG: (2-(N-(7-Nitrobenz-2-oxa-1,3-diazol-4-yl) Amino)-2-Deoxyglucose
2-DG: 2-Deoxy-D-glucose
ECAR: Extracellular acidification rate
FCCP: Carbonyl cyanide-4(trifluoromethoxy) phenylhydrazone
OCR: Oxygen consumption rate
ANOVA: Analysis of variance
MTT: 3-(4,5-Dimethylthiazol-2-yl)-2,5-diphenyltetrazolium bromide
ERK: Extracellular signal regulated kinase
AKT: Serine/threonine protein kinase
MCT: Monocarboxylate transporter
LDHA: Lactate dehydrogenase A
LDLR: Low-density lipoprotein receptor
OS: Overall survival
RFS: Relapse-free survival
PPS: Post progression survival
