# Correction: Cholesterol reprograms glucose and lipid metabolism to promote proliferation in colon cancer cells

**DOI:** 10.1186/s40170-023-00321-3

**Published:** 2023-10-26

**Authors:** Shyamananda Singh Mayengbam, Abhijeet Singh, Himanshi Yaduvanshi, Firoz Khan Bhati, Bhavana Deshmukh, Dipti Athavale, Pranay L. Ramteke, Manoj Kumar Bhat

**Affiliations:** National Centre for Cell Science, Department of Biotechnology, Government of India, Savitribai Phule Pune University Campus, Ganeshkhind, Pune, 411 007 India

**Correction: Cancer & Metabolism 11, 15 (2023)**

**https://doi.org/10.1186/s40170-023-00315-1**

Following publication of the original article [1], it came to the author's attention that there were two errors in the figures and supplementary files of the article: 1) In Fig. 6, panel D, the MCT-2 blot in the presence of LDL and HDL had been inadvertently duplicated; for ESM 2, the wrong file (‘Supplementary figure blots (uncut)’ instead of ‘Uncut blot of main figures’) had been provided. The files have since been corrected in the published article. The authors thank you for reading and apologize for any inconvenience caused.
