# Supplementary material for: Cholesterol reprograms glucose and lipid metabolism to promote proliferation in colon cancer cells
Source: Cancer Metab. 2023 Sep 13;11:15. doi: 10.1186/s40170-023-00315-1 (PMC10500936; doi:10.1186/s40170-023-00315-1)

**Cholesterol reprograms glucose and lipid metabolism to promote proliferation in colon cancer cells**

**Authors:** Shyamananda Singh Mayengbam, Abhijeet Singh, Himanshi Yaduvanshi, Firoz Khan Bhati, Bhavana Deshmukh, Dipti Athavale, Pranay L. Ramatake, and Manoj Kumar Bhat^*^.

**Authors' affiliations:**

National Centre for Cell Science, Department of Biotechnology, Government of India, Savitribai Phule Pune University campus, Ganeshkhind, Pune 411 007, India.

***Corresponding author:** Dr. Manoj Kumar Bhat, National Centre for Cell Science, Savitribai Phule Pune University, Ganeshkhind, Pune-411 007, India. Tel.: +91-20-25708066; Fax: +91-20-25692259. E-mail address: manojkbhat@nccs.res.in.

**I.**

**
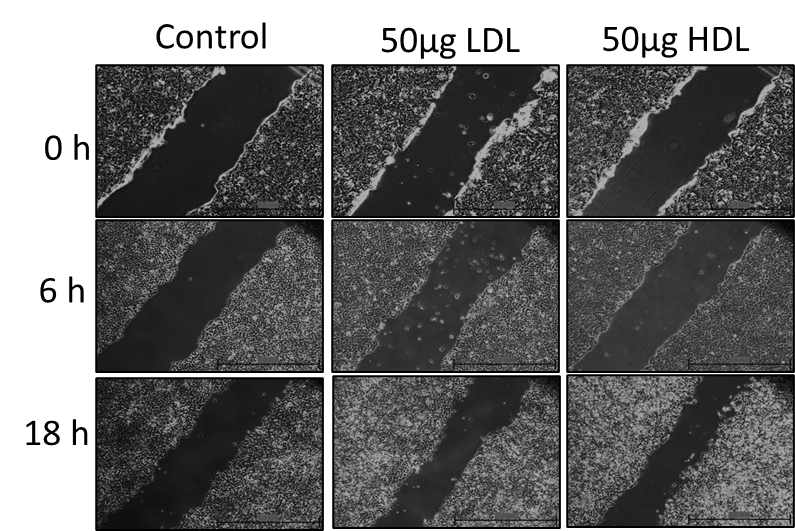
**

**Supplementary figure 1. Effect of LDLc and HDLc supplementation on the proliferation of colon cancer cells. (A-D)** Colon cancer cells were cultured for 10-12 days in 1% FBS-containing media without or with LDLc (50 µg/ml) or HDLc (50µg/ml) and allowed to form colonies. Images of 0.05% crystal violet-stained colonies of colon cancer cells without or with LDLc or HDLc were taken using an Olympus DSLR camera. Quantification was done using Image J software. The bar graph represented the percent colony formed with respect to untreated controls in (A & B) HT-29, (C & D) Caco-2, and (E & F) HCT-116 p53 -/- and HCT-15 cells. (G & H) MTT assay for cell proliferation in HCT-116 cells treated with different concentrations of LDLc and HDLc for 48 h. Experiments were done in triplicate and performed twice. (I) Scratch assay was performed using HCT-116 cells exposed to vehicle control or 50 µg/ml of LDLc or HDLc and pictures were taken at different time points. Independent experiment was performed twice. Pictures and data is presented from an experiment.

**
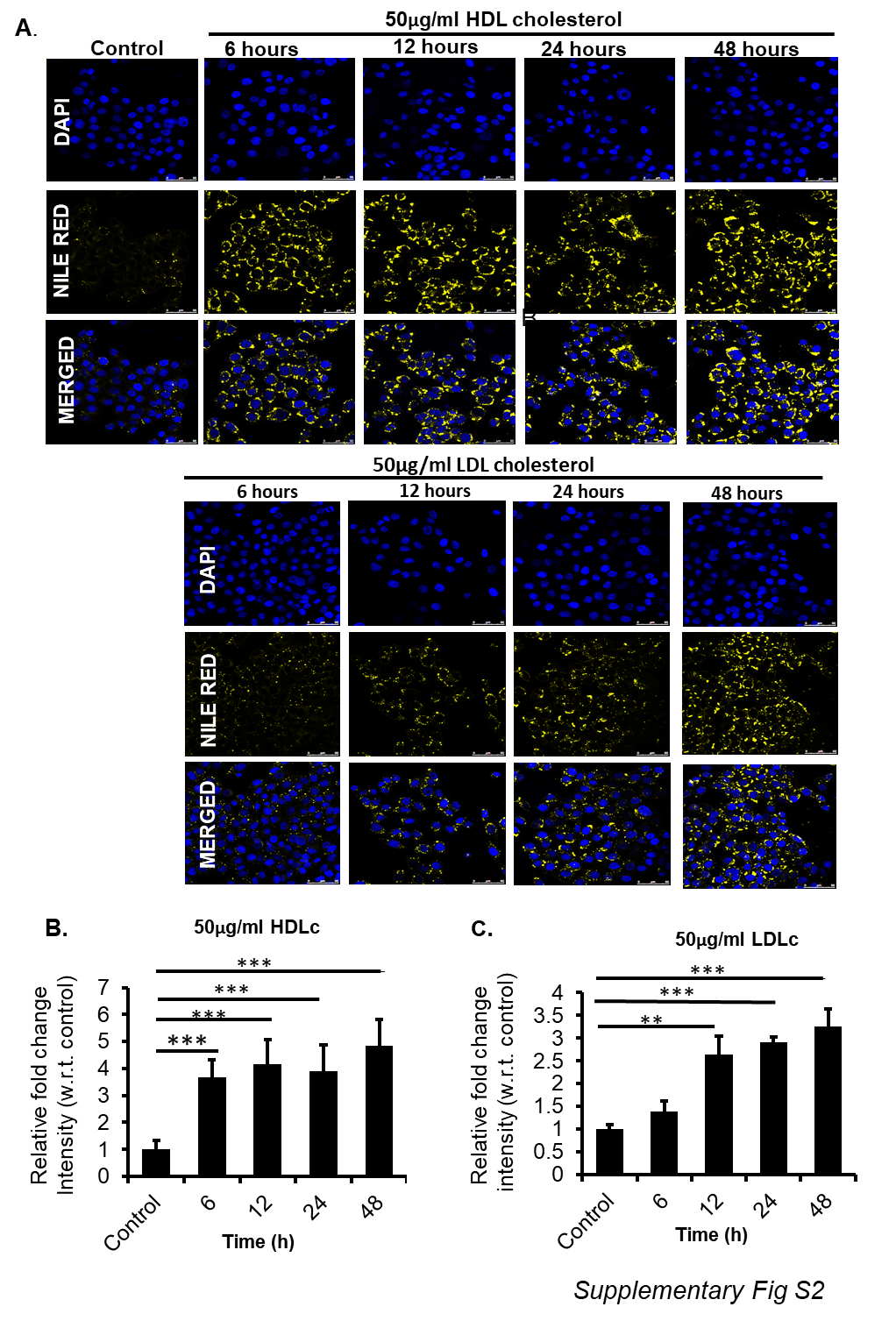
**

**Supplementary figure 2. Time-dependent lipid accumulation by colon cancer cells upon LDLc or HDLc treatment.** HCT-116 cells were treated with vehicle or 50 µg/ml of LDLc/HDLc for different time points. Cells were stained with Nile red, followed by the analysis of lipid accumulation (Nile red staining) by Confocal imaging (A) Image showing Nile red staining after HDLc and LDLc treatment respectively. (B) & (C) Relative intensity of Nile red staining in cells treated with HDLc and LDLc respectively for different time points.

**
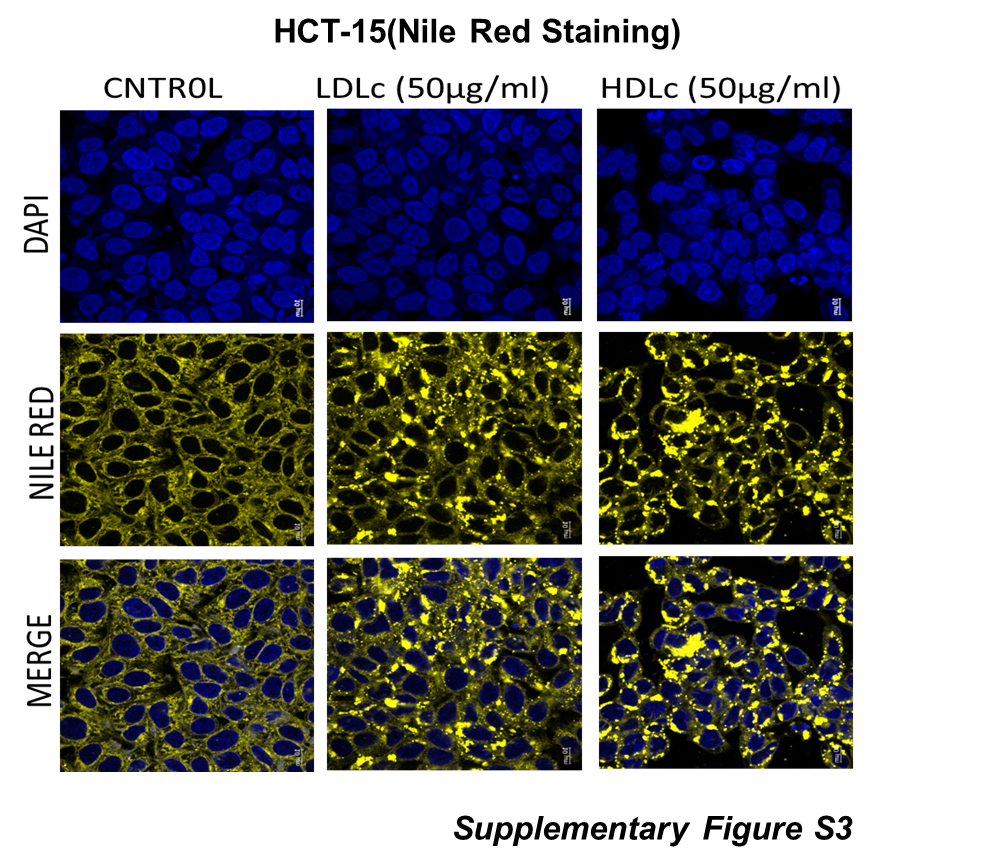
**

**Supplementary figure 3. Lipid accumulation upon LDLc and HDLc treatment in HCT-15 cells.** HCT-15 cells were treated with vehicle or 50 µg/ml of LDLc/HDLc for 48 h followed by quantification of lipid accumulation (Nile red staining) by Confocal imaging. The experiment was done in triplicate.


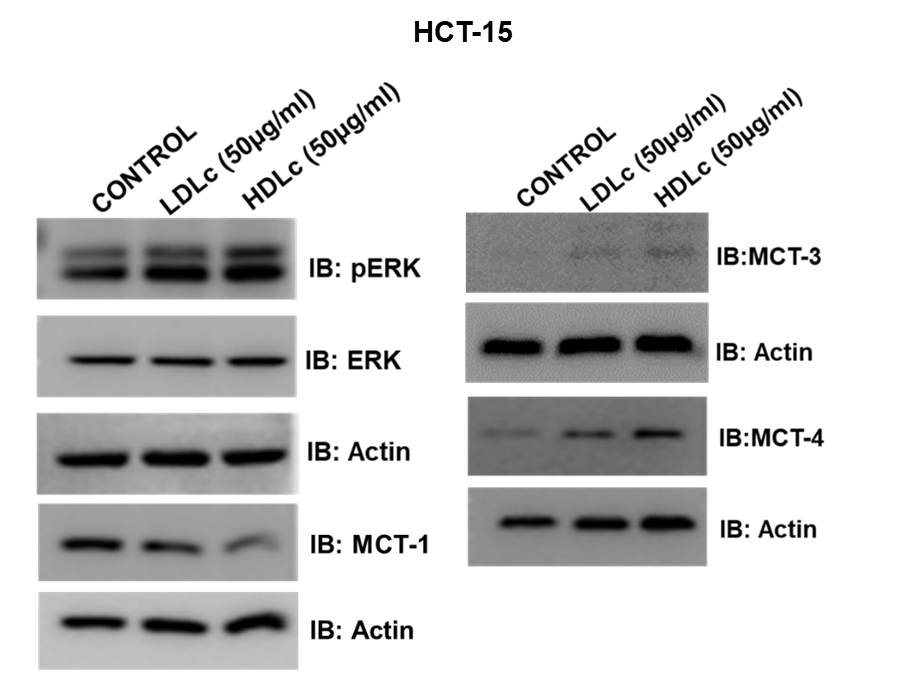


**Supplementary figure 4. Immunoblot analysis of molecules associated with colorectal cancer cells proliferation after LDLc or HDLc treatment.** HCT-15 cells were exposed to vehicle control or 50 µg/ml of LDLc or HDLc for 48 h, and immunoblotting for pERK, ERK, MCT-1, and MCT-3 was performed from the whole cell lysate of HCT-15. Actin was used as a loading control.


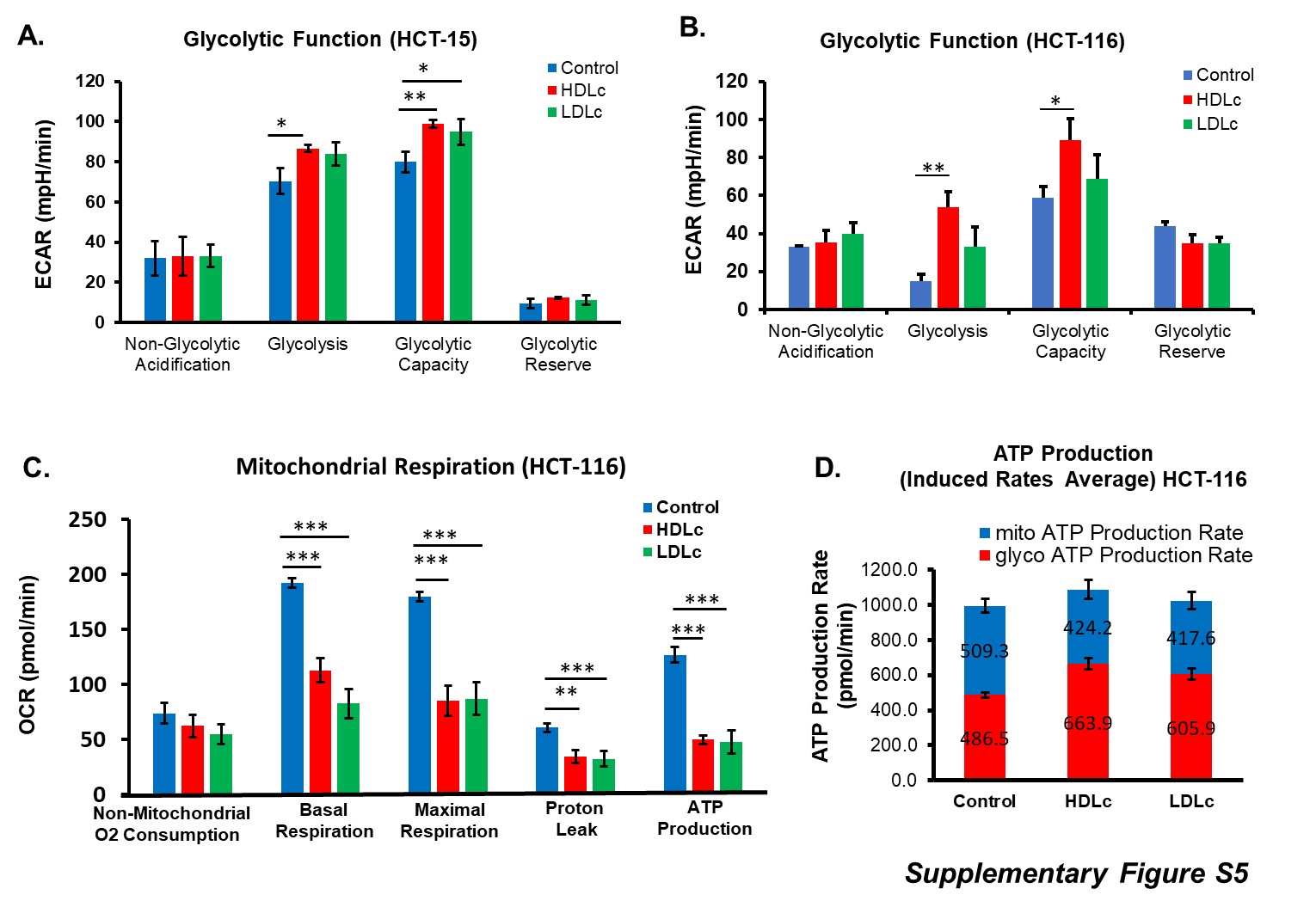


**Supplementary figure 5. Role of LDLc and HDLc in glycolytic function, mitochondrial respiration, and ATP production in colon cancer cells.** HCT-116/HCT-15 cells were pre-treated with 50 μg/ml LDLc or HDLc for 12 h in 1% FBS-containing media followed by the analysis of various metabolic parameters by Seahorse XFe24 Analyzer. (A) & (B) Glycolytic stress (ECAR) function analysis in HCT-15 and HCT-116 respectively showing different glycolytic stress parameters (C) Graph showing different parameters of mitochondrial respiration (OCR) in HCT-116 cells. (D) Seahorse XeF real-time ATP rate analysis of HCT-116 cells upon LDLc and HDLc treatment. Bar graph showing ATP production rate in cells treated with vehicle control LDLc or HDLc. The experiment was done in triplicate.

**
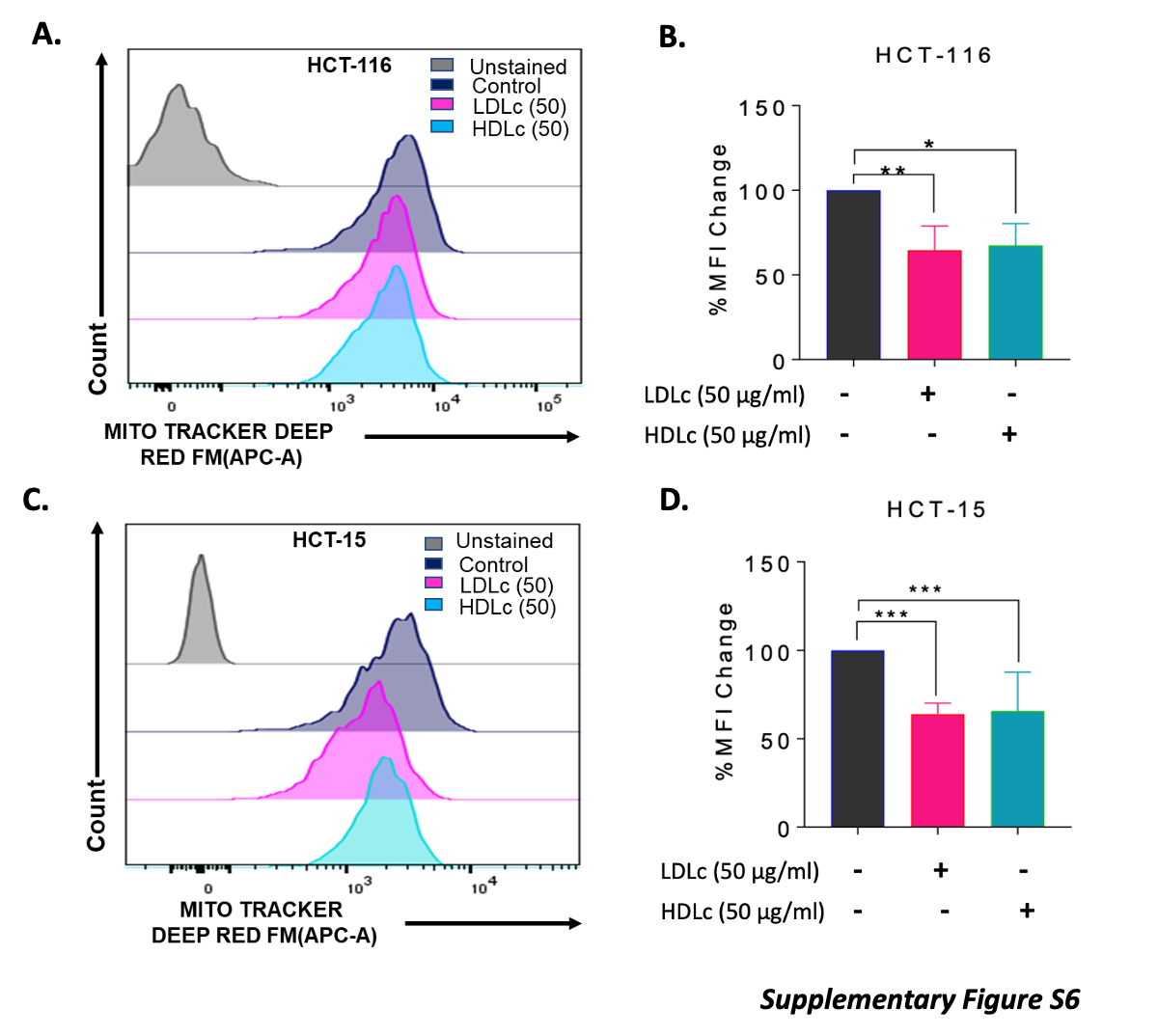
**

**Supplementary figure 6. Role of LDLc and HDLc in the mitochondrial biogenesis in colon cancer cells.** HCT-116 and HCT-15 cells were treated with 50 μg/ml of LDLc / HDLc or vehicle for 48 h as mentioned in the method section and stained with Mito tracker deep red and analysed through flow cytometry. Histogram and MFI bar showing different mitochondrial densities in HCT-116 cells (A & B) and HCT-15 cells (C) & (D). Experiments were done twice in triplicates.


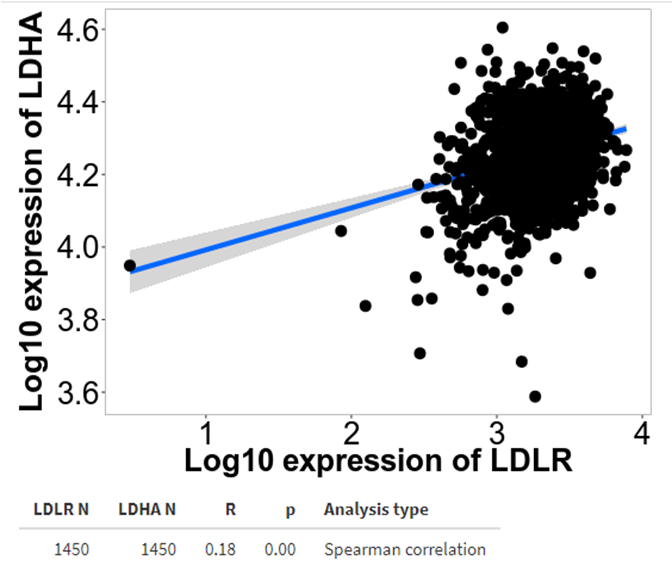


**Supplementary figure 7.1: Correlation between LDLR and LDHA gene expression in colon cancer patient. Data was extracted using TMNplotter**

**
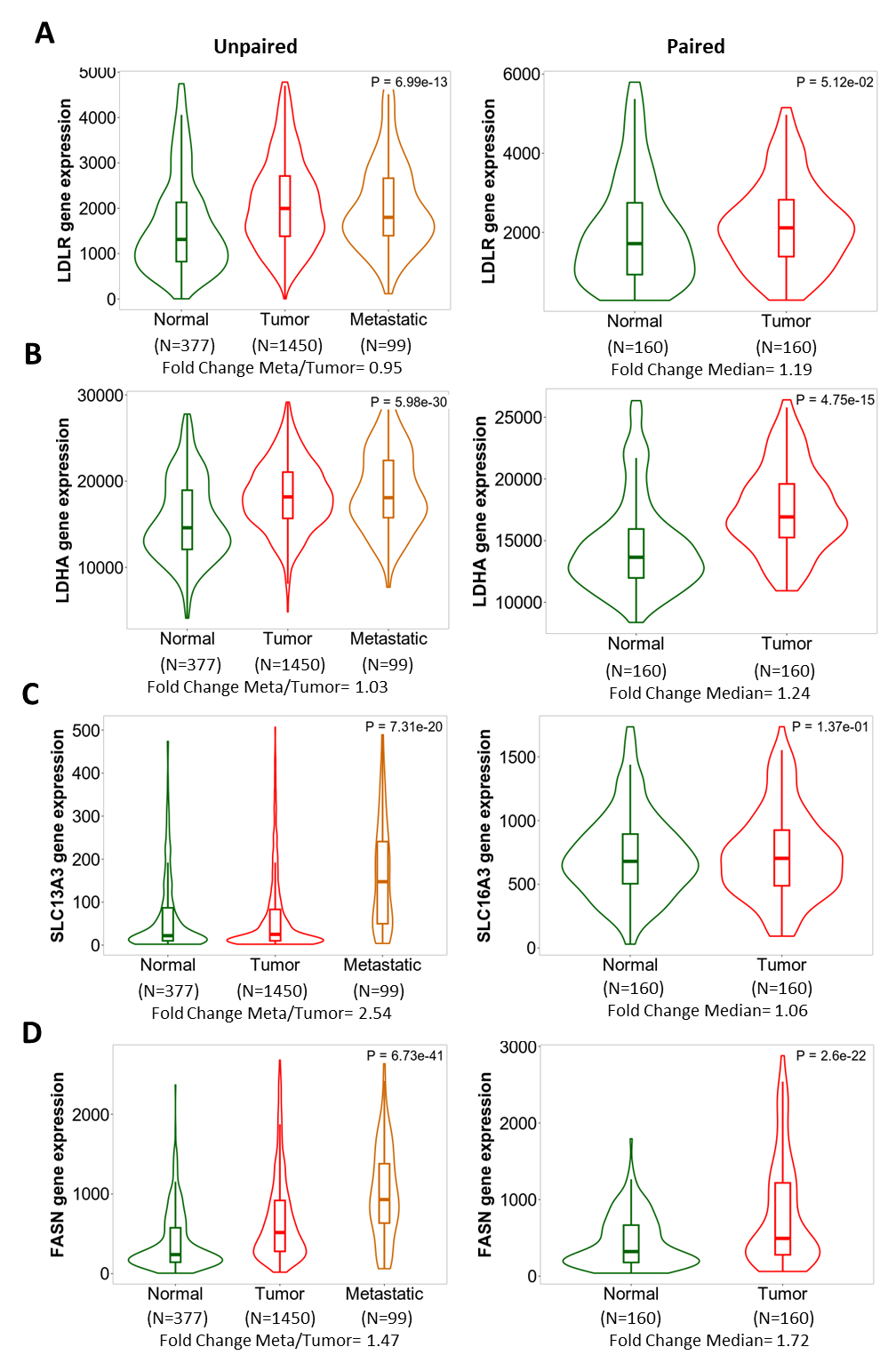
**

**Supplementary figure 7.2:** **Gene expression profile of LDLR, LDHA, MCT-4, and FASN in tumor, non-tumor and metastatic tissue of human colon from Gene CHIP Data**. **(A):** Unpaired and paired gene expression profile of LDLR. **(B):** Unpaired and paired gene expression profile of LDHA. **(C):** Unpaired and paired gene expression profile of MCT-4. **(D):** Unpaired and paired gene expression profile of FASN.

**
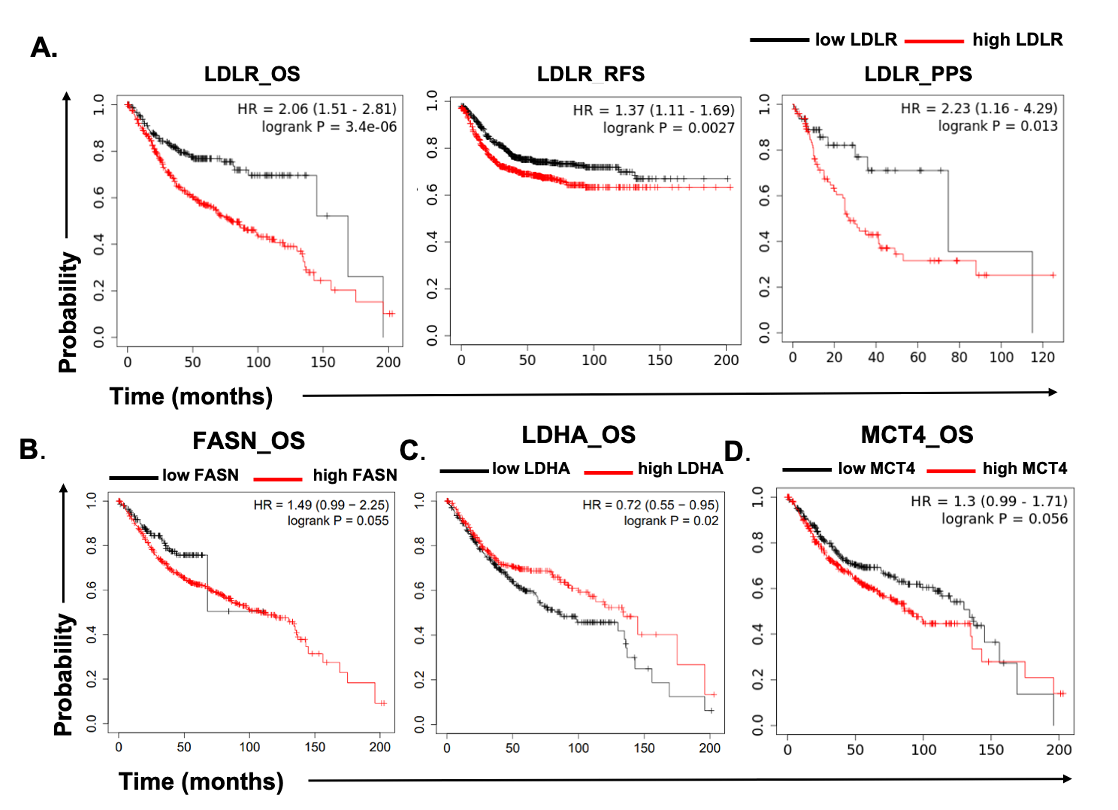
**

**Supplementary figure 7.3:** **Kaplan-Meier curves for survival of colon cancer patients by LDLR, FASN, LDHA, and MCT4 status.** **(A):**  Overall survival, Relapse free survival, and Post progression survival by LDLR high and low status **(B):**  Overall survival by FASN high and low status. **(C)** Overall survival by LDHA high and low Status **(D)** Overall survival by MCT4 high and low status


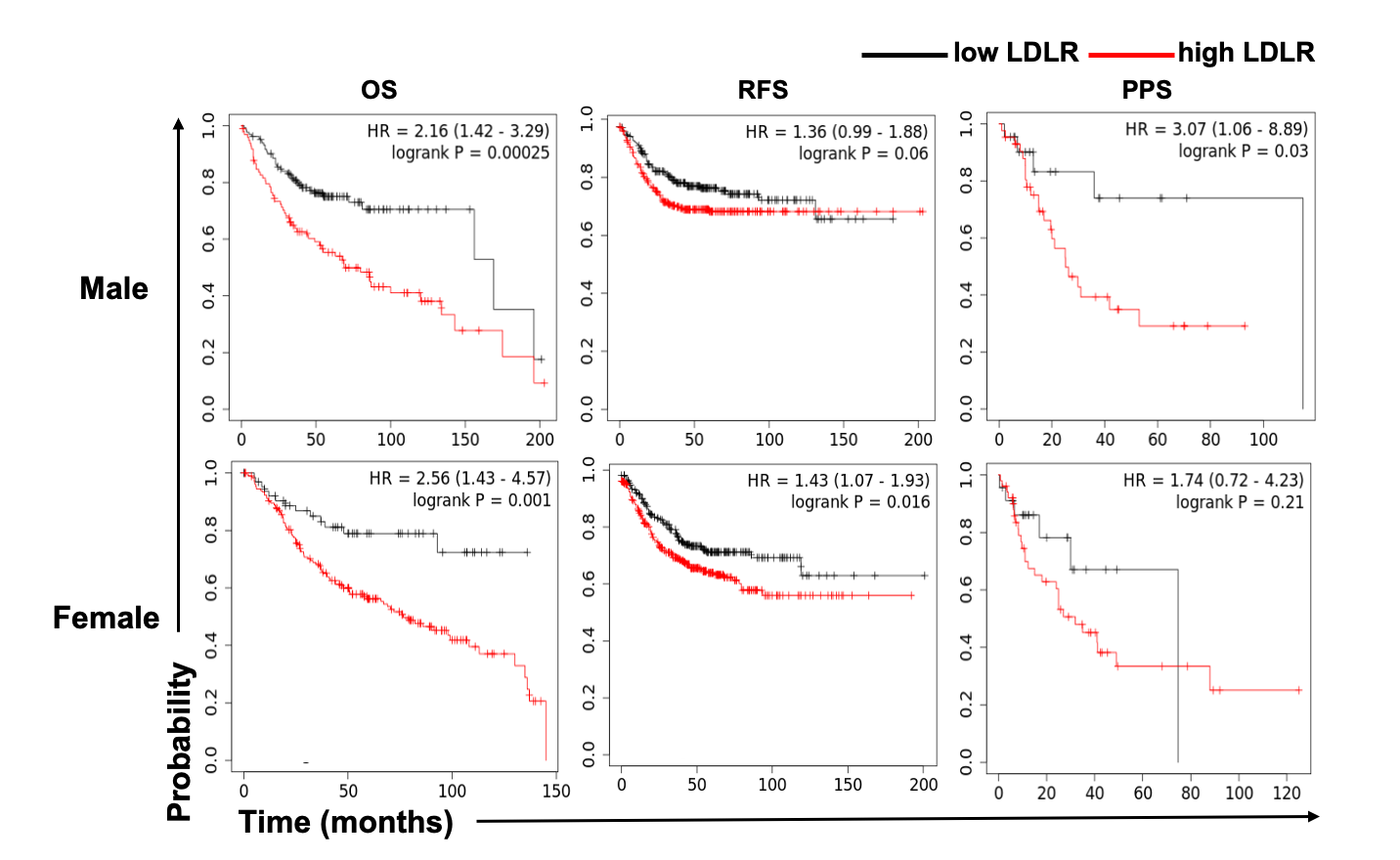


**Supplementary figure 7.4:** **Kaplan-Meier curves for Overall survival, Relapse free survival, and Post progression survival of colon cancer patients in male and female by high and low LDLR expression.**


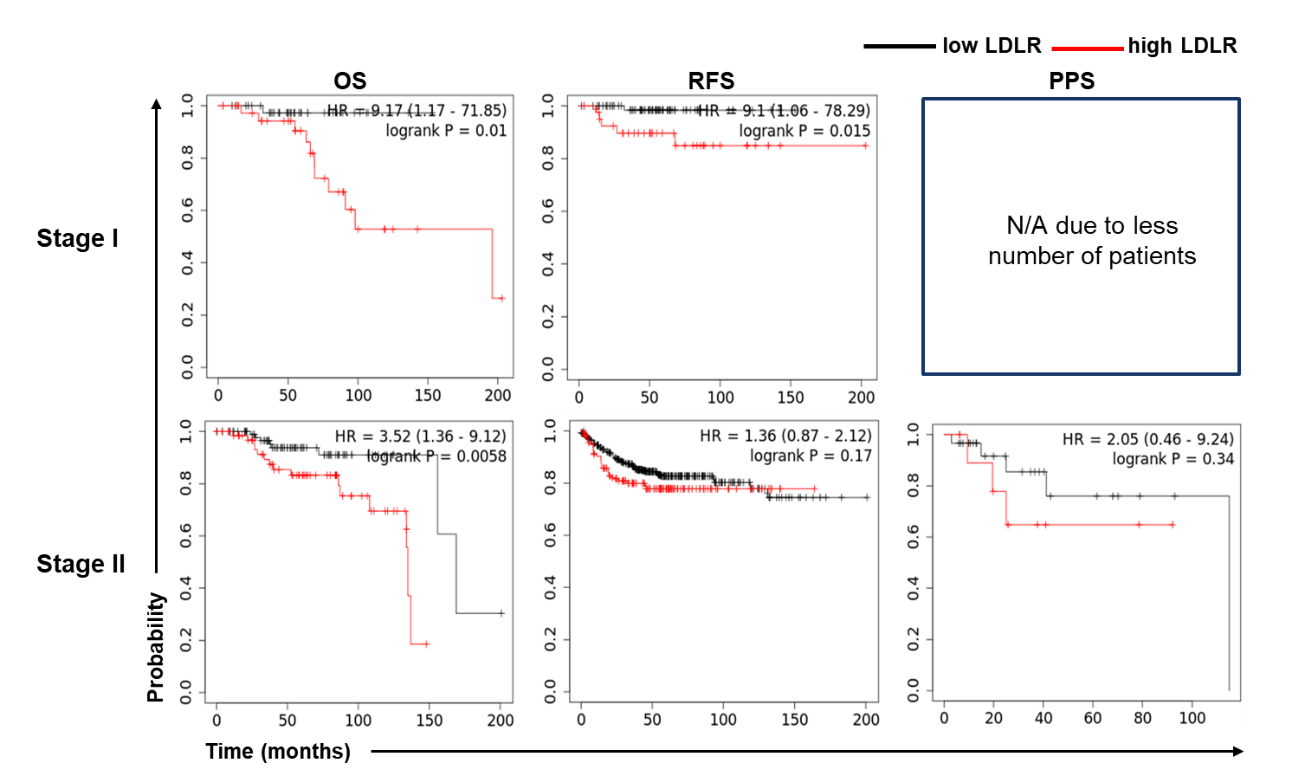


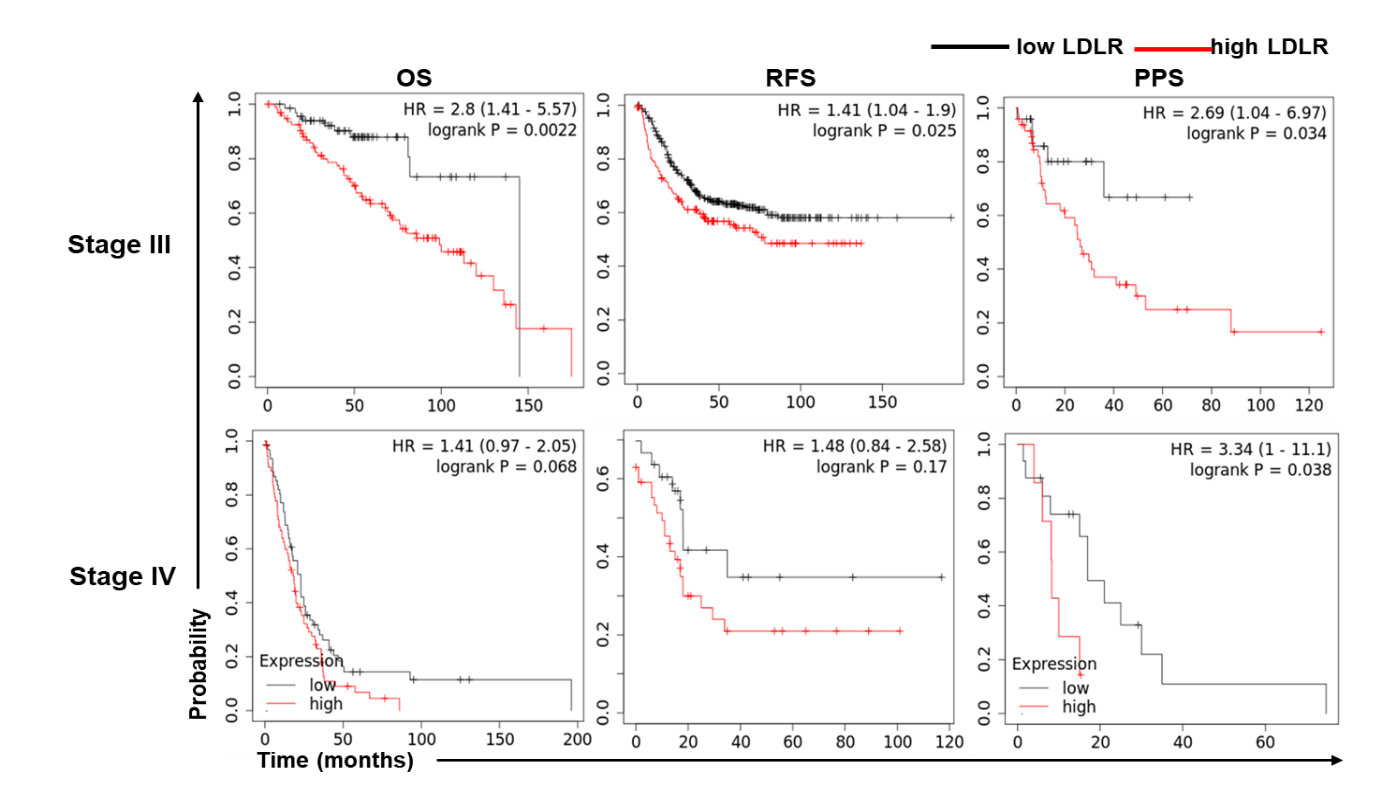


**Supplementary figure 7.5:** **Kaplan-Meier curves for Overall survival, Relapse free survival, and Post progression survival of colon cancer patients in different stages (I-IV) by high and low LDLR expression.**


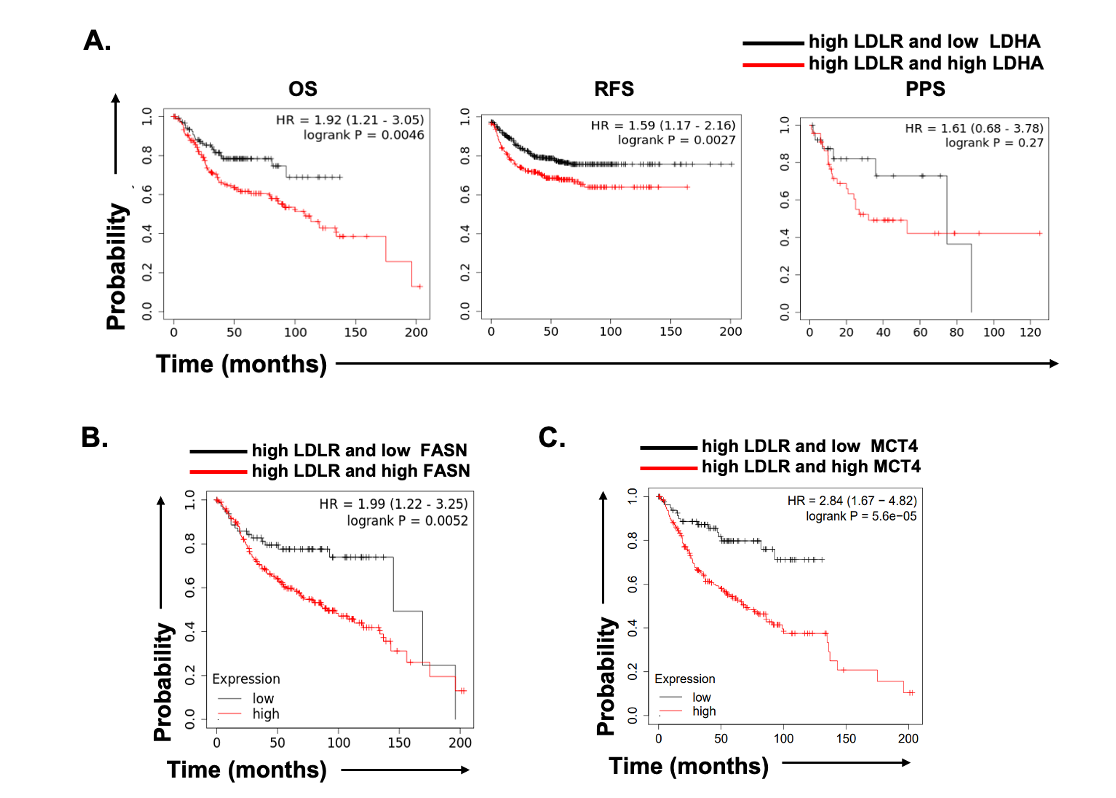


**Supplementary figure 7.6:** **Kaplan-Meier curves for survival of colon cancer patients with high LDLR expression together with high or low LDHA, FASN, and MCT4 expression**. (**A):** Overall survival, Relapse free survival, and Post progression survival by LDLR high with low LDHA and LDLR high with high LDHA status. **(B):**  Overall survival, survival by LDLR high with low FASN and LDLR high with high FASN status. **(D):** Overall survival survival by LDLR high with low MCT4 and LDLR high with high MCT4 status


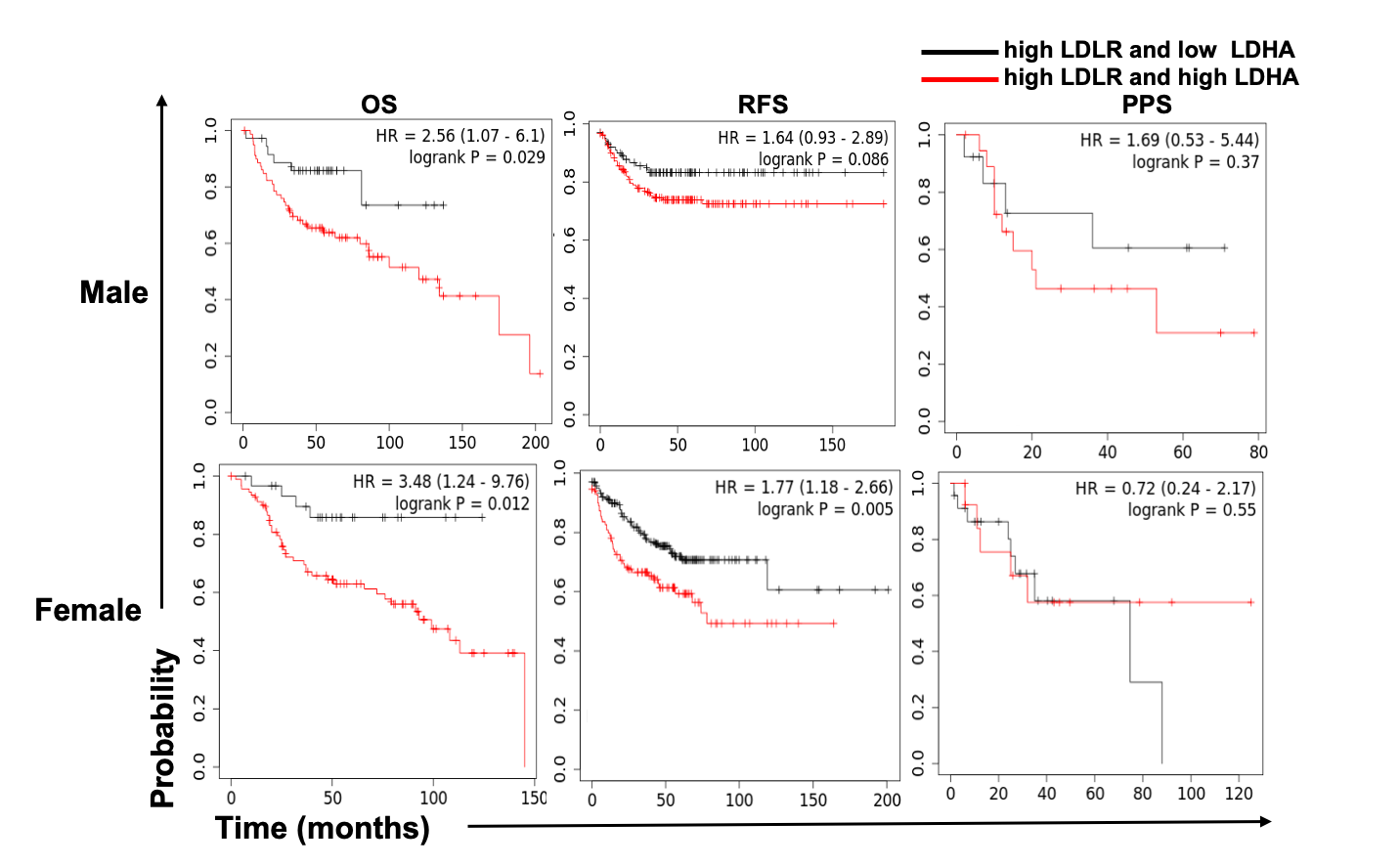


**Supplementary figure 7.7:** **Kaplan-Meier curves for Overall survival, Relapse free survival, and Post progression survival of colon cancer patients in male and female by high LDLR with low LDHA expression in comparison to high LDLR with high LDHA expression.**


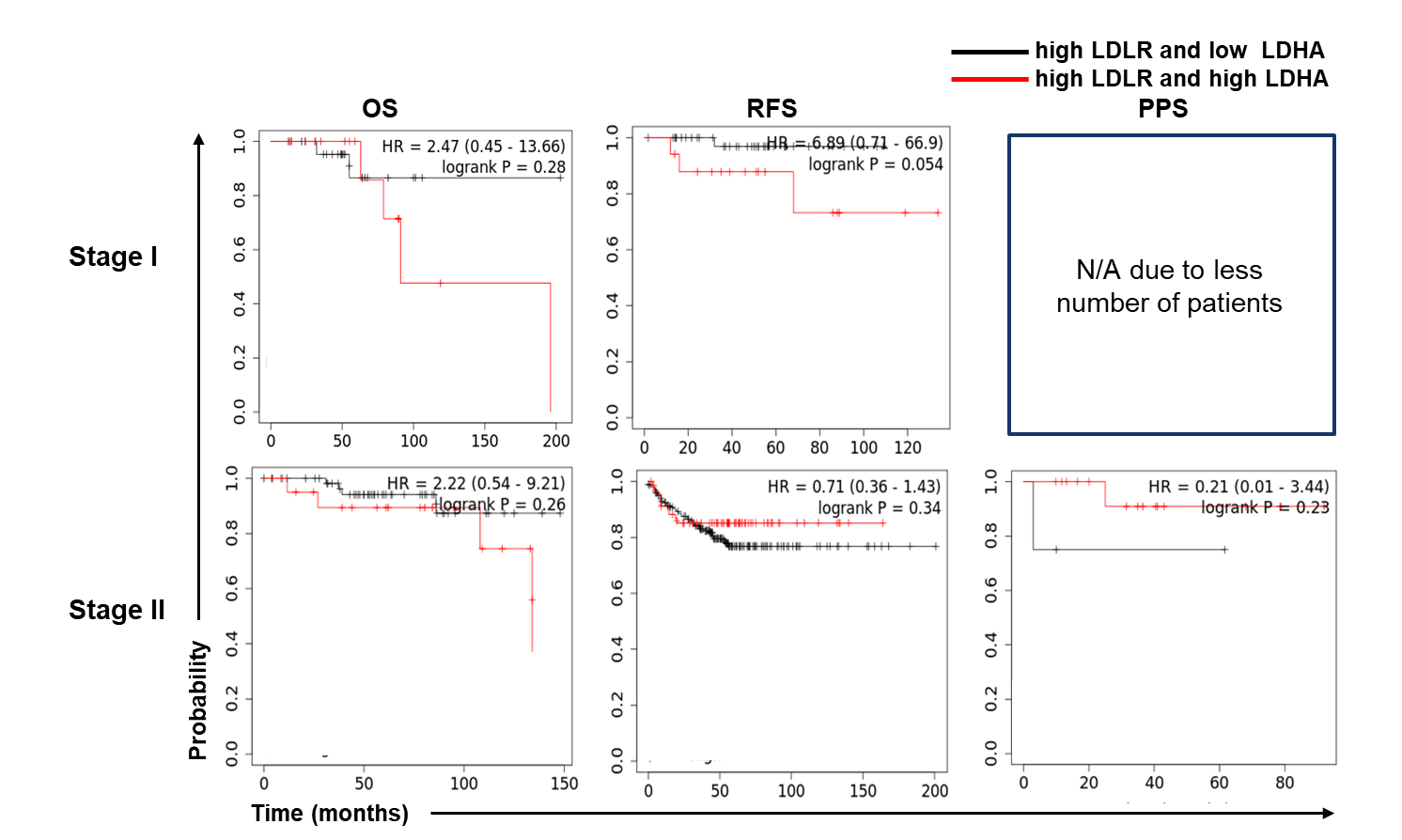


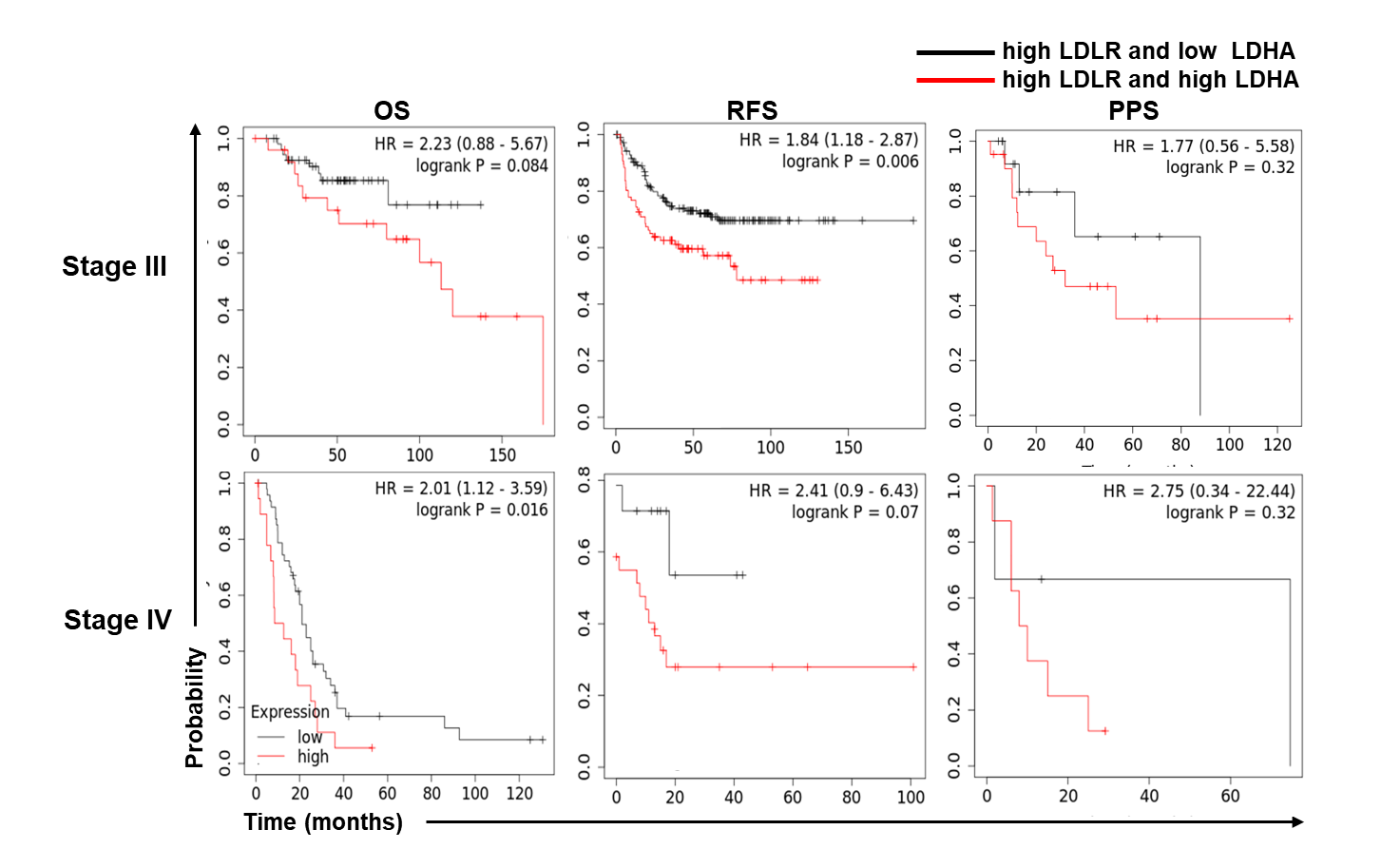


**Supplementary figure 7.8:** **Kaplan-Meier curves for Overall survival, Relapse free survival, and Post progression survival of colon cancer patients in different stages (I-IV) by High LDLR with low LDHA expression in comparison to High LDLR with high LDHA expression.**

**
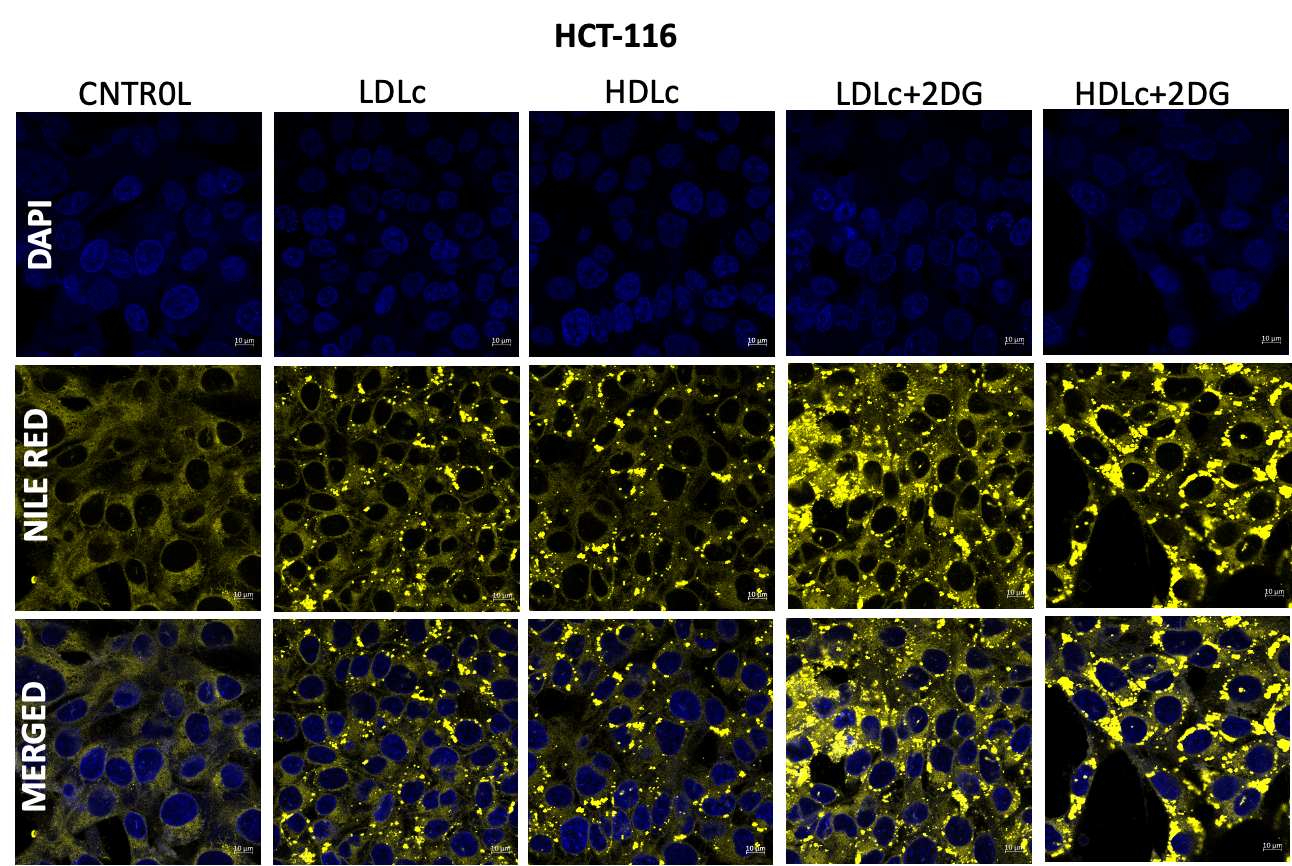
**

**Supplementary figure 8: Effect of 2-DG (2 Deoxy Glucose) on lipid accumulation in cells treated with LDLc and HDLc.** HCT-116 cells were treated with vehicle, 50µg/ml of LDLc/HDLc with or without 2-DG for 24 h and stained with Nile red, followed by the analysis of lipid accumulation (Nile red staining) by Confocal imaging.


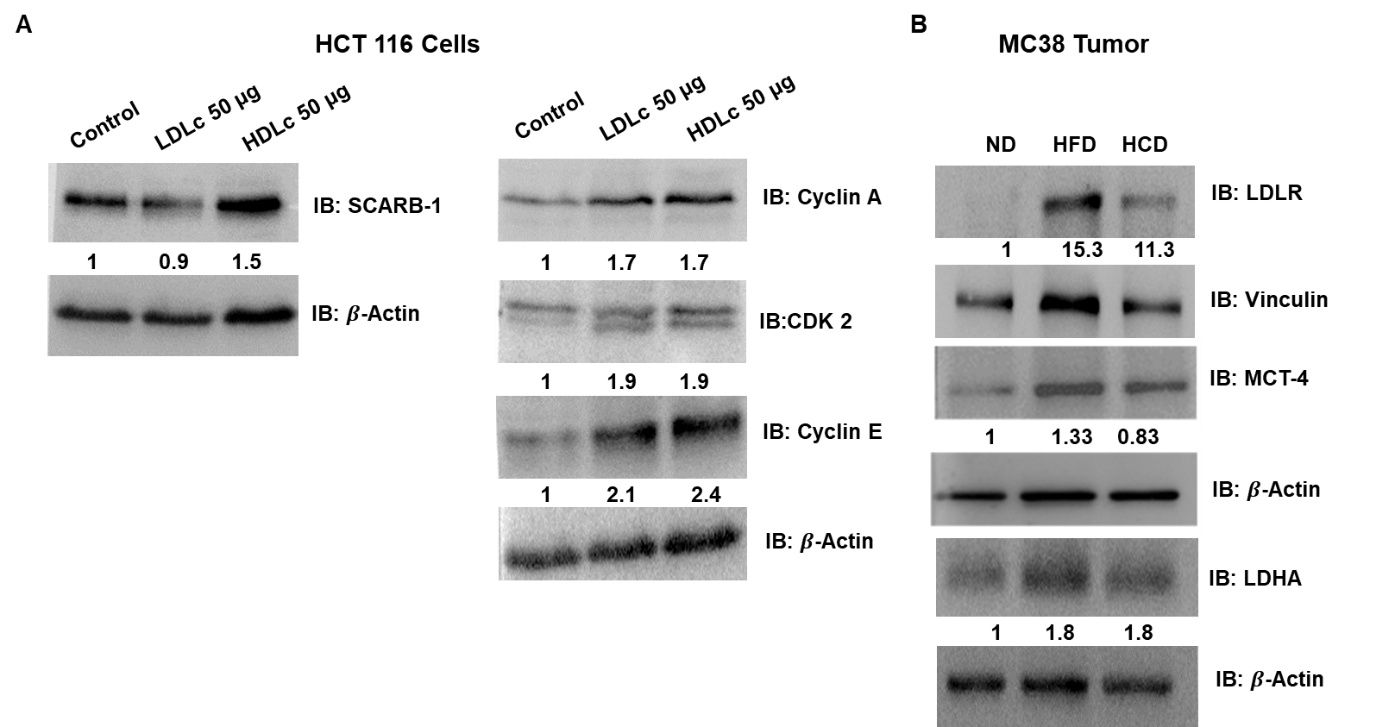


**Supplementary figure 9: Immunoblot analysis in lysates of HCT-116 cells and MC-38 tumor.** (A) HCT-116 cells were exposed to vehicle control or 50µg/ml of LDLc or HDLc for 24 h, and immunoblotting for SCARB-1, Cyclin A , CDK 2 and Cyclin E was performed from the whole cell lysate of HCT-116. Actin was used as a loading control. (B) Immunoblot analysis of LDLR, LDHA, and MCT-4 was performed in lysates of MC-38 tumor tissue obtained from ND, HFD, and HCD mice. Actin and Vinculin were used as a loading control. Quantification of immunoblot were performed by using NIH image J software.

**Supplementary table 1: Serum parameters changes in ND, HFD, and HCD pooled mice serum**

| **S. No.** | **Serum Parameter** | **ND Mice** | **HFD Mice** | **HCD mice** |
| --- | --- | --- | --- | --- |
| **1** | Cholesterol (mg/dl) | 61.45 $\pm$ 18 | 102.34$\pm$ 10 | 112.09 $\pm$ 21 |
| **2** | Triglyceride (mg/dl) | 43.30 $\pm$ 5..34 | 52.72 $\pm$ 9.7 | 55.5 $\pm$ 9.4 |
| **3** | Adiponectin (ng/ml) | 131.22 $\pm$ 2.94 | 124.59 $\pm$ 3.51 | 123.9 $\pm$ 1.77 |
| **4** | Leptin (pg/ml) | 3573 $\pm$ 641 | 7490 $\pm$ 605 | 4683 $\pm$ 71 |

**Supplementary table 2: High expression of LDLR results in poor prognosis of colon cancer patient**


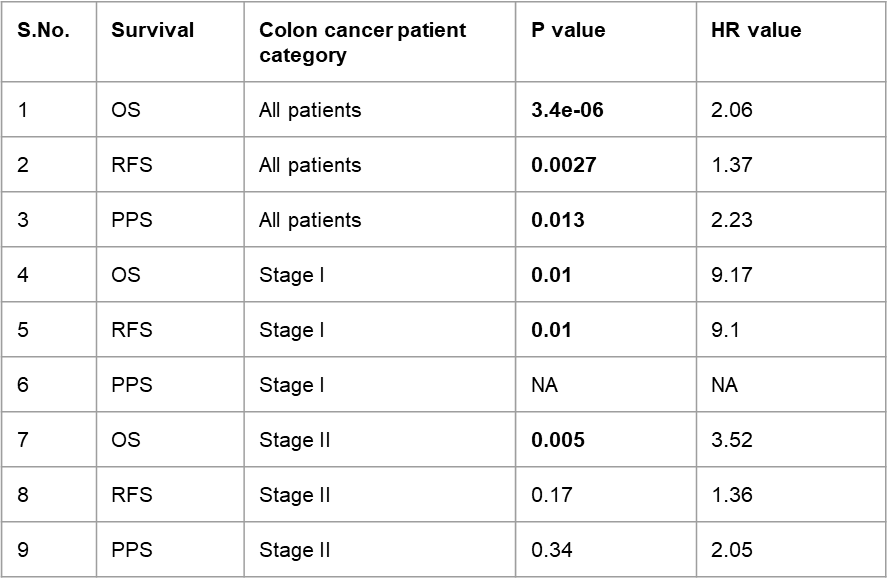


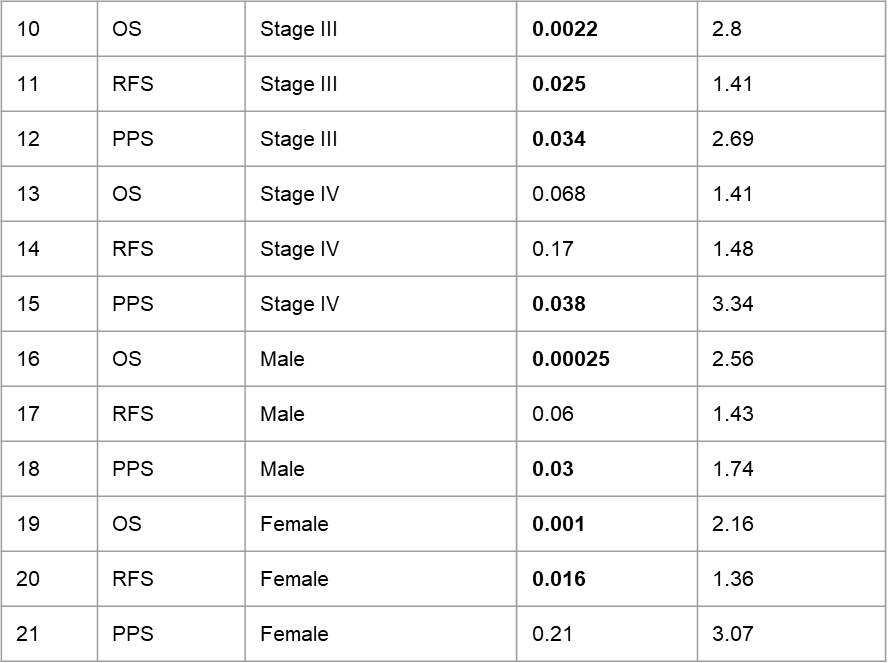


**Supplementary table 3: Survival data of colon cancer patients expressing high LDLR with low or high LDHA expression**


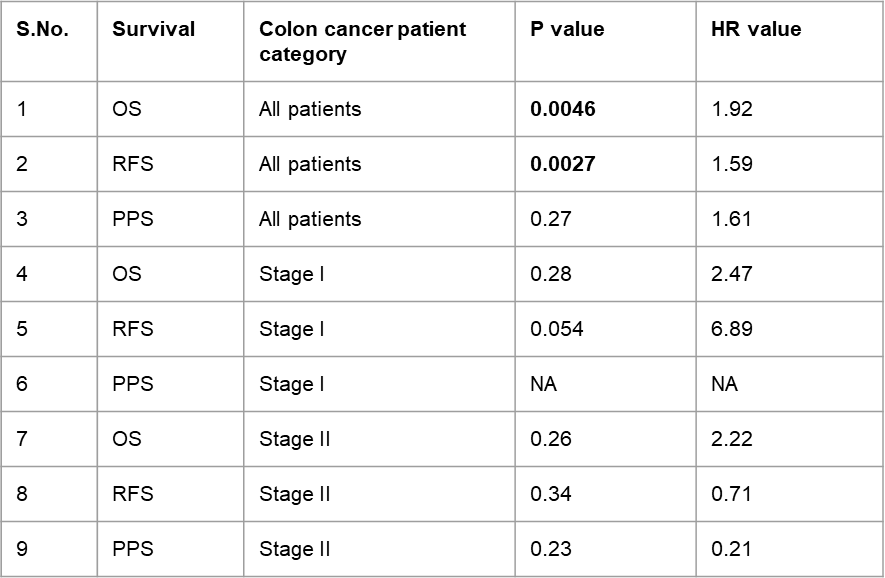


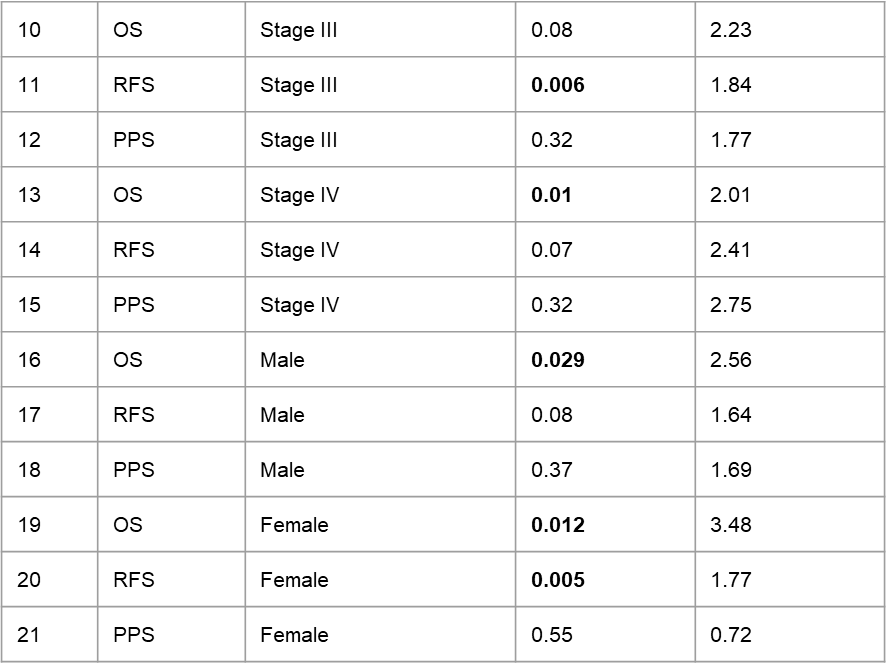

Supplement: Supplementary file 1 — Additional file 1: Supplementary figure 1. Effect of LDLc and HDLc supplementation on the proliferation of colon cancer cells. Supplementary figure 2. Time-dependent lipid accumulation by colon cancer cells upon LDLc or HDLc treatment. Supplementary figure 3. Lipid accumulation upon LDLc and HDLc treatment in HCT-15 cells. Supplementary figure 4. Immunoblot analysis of molecules associated with colorectal cancer cells proliferation after LDLc or HDLc treatment. Supplementary figure 5. Role of LDLc and HDLc in glycolytic function, mitochondrial respiration, and ATP production in colon cancer cells. Supplementary figure 6. Role of LDLc and HDLc in the mitochondrial biogenesis in colon cancer cells. Supplementary figure 7.1. Correlation between LDLR and LDHA gene expression in colon cancer patients. Data was extracted using TMNplotter. Supplementary figure 7.2. Gene expression profile of LDLR, LDHA, MCT-4, and FASN in tumor, non-tumor and metastatic tissue of human colon from Gene CHIP Data. Supplementary figure 7.3. Kaplan-Meier curves for survival of colon cancer patients by LDLR, FASN, LDHA, and MCT4 status. Supplementary figure 7.4. Kaplan-Meier curves for Overall survival, Relapse free survival, and Post progression survival of colon cancer patients in male and female by high and low LDLR expression. Supplementary figure 7.5. Kaplan-Meier curves for Overall survival, Relapse free survival, and Post progression survival of colon cancer patients in different stages (I-IV) by high and low LDLR expression. Supplementary figure 7.6. Kaplan-Meier curves for survival of colon cancer patients with high LDLR expression together with high or low LDHA, FASN, and MCT-4 expression. Supplementary figure 7.7. Kaplan-Meier curves for Overall survival, Relapse free survival, and Post progression survival of colon cancer patients in male and female by high LDLR with low LDHA expression in comparison to high LDLR with high LDHA expression. Supplementary figure 7.8. Kaplan-Meier curv [file 40170_2023_315_MOESM1_ESM.docx]
