# Supplementary material for: Cholesterol reprograms glucose and lipid metabolism to promote proliferation in colon cancer cells
Source: Cancer Metab. 2023 Sep 13;11:15. doi: 10.1186/s40170-023-00315-1 (PMC10500936; doi:10.1186/s40170-023-00315-1)
Supplement: Supplementary file 2 — Additional file 2. [file 40170_2023_315_MOESM2_ESM.pptx]

## Slide 1
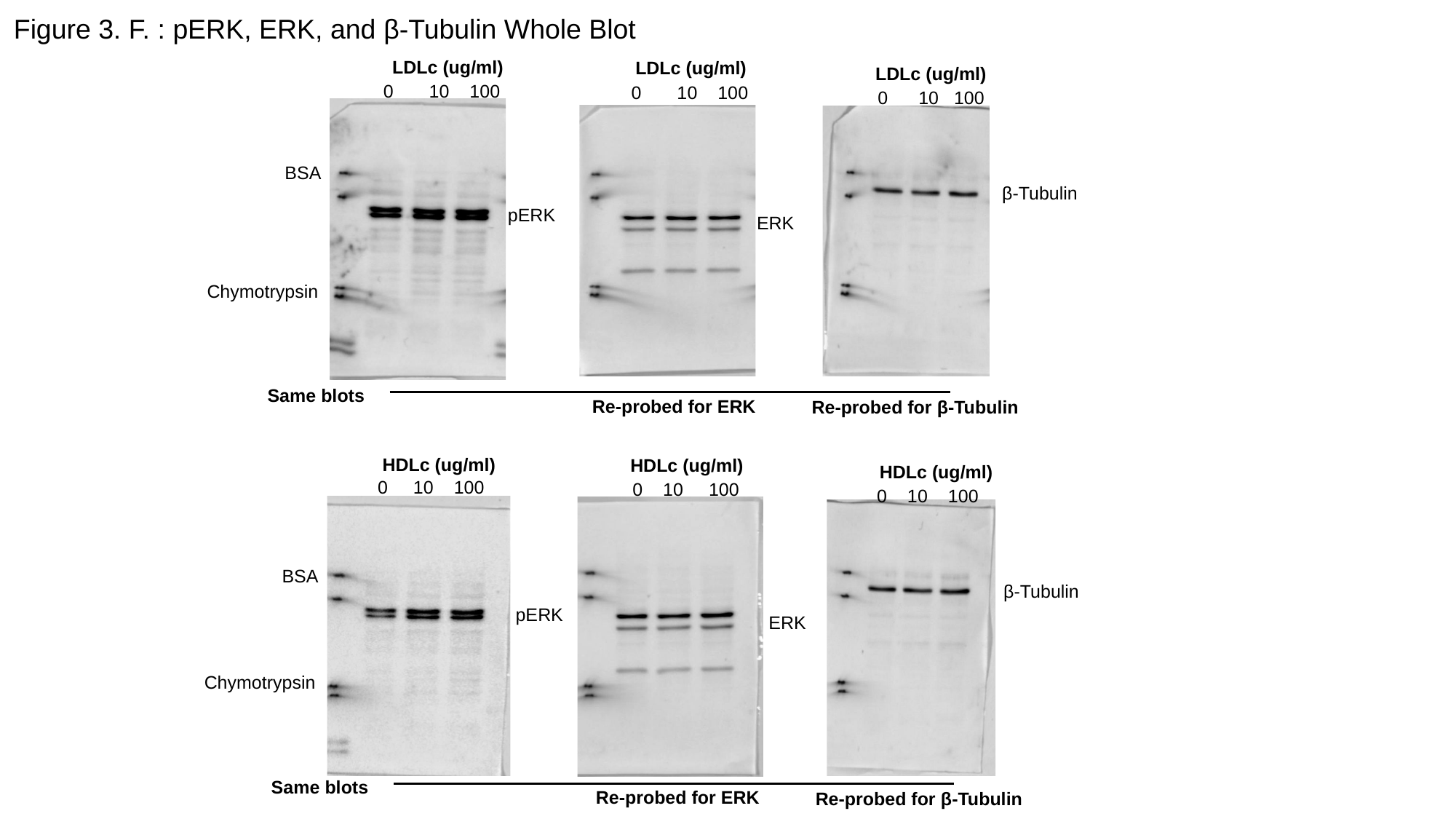

Figure 3. F. : pERK, ERK, and β-Tubulin Whole Blot
LDLc (ug/ml)
 0 10 100
BSA
pERK
Chymotrypsin
LDLc (ug/ml)
 0 10 100
ERK
LDLc (ug/ml)
 0 10 100
β-Tubulin
Same blots
Re-probed for ERK
Re-probed for β-Tubulin
HDLc (ug/ml)
 0 10 100
pERK
HDLc (ug/ml)
 0 10 100
ERK
BSA
Chymotrypsin
HDLc (ug/ml)
 0 10 100
β-Tubulin
Same blots
Re-probed for ERK
Re-probed for β-Tubulin

## Slide 2
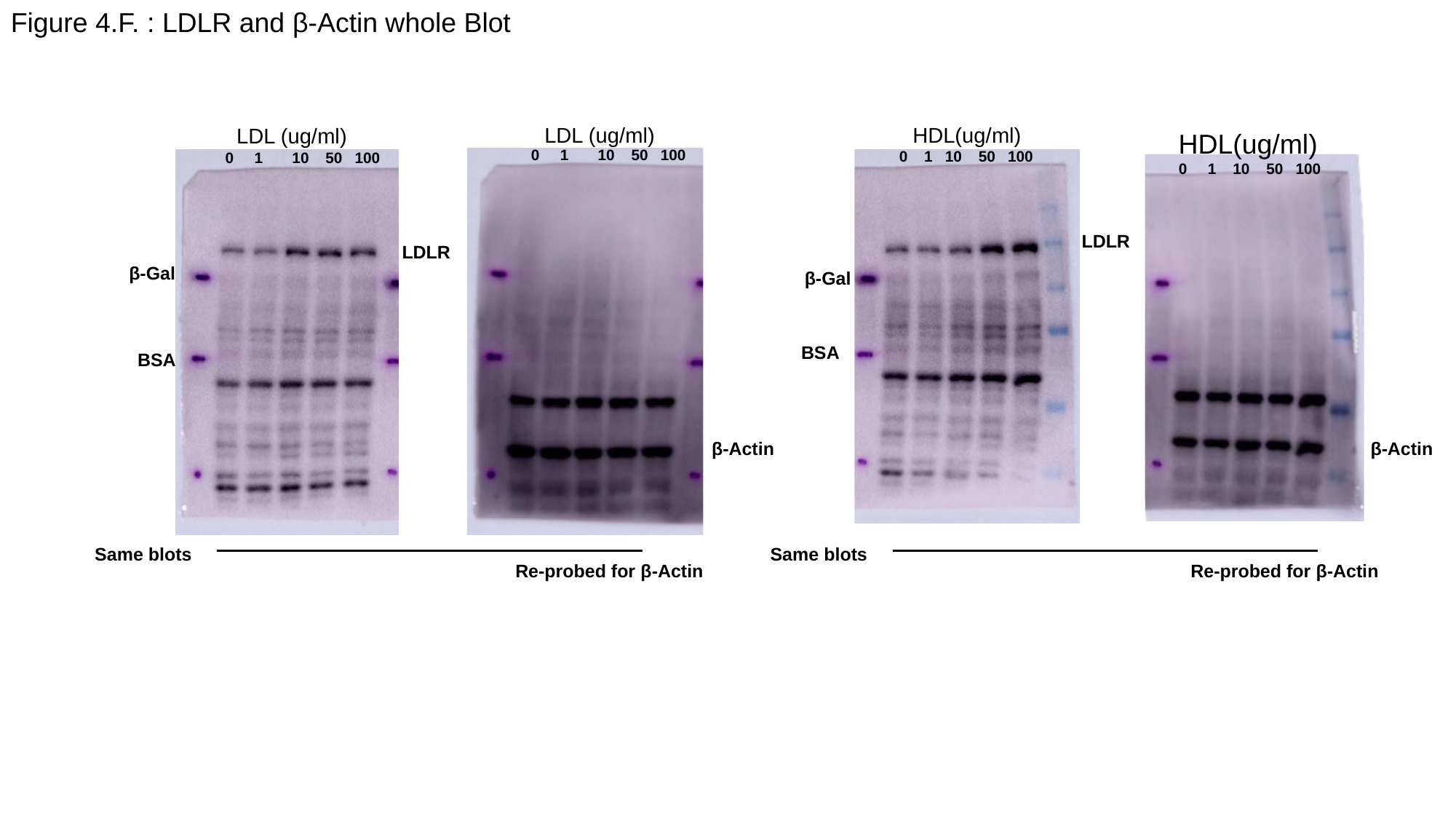

Figure 4.F. : LDLR and β-Actin whole Blot
HDL(ug/ml)
HDL(ug/ml)
 0 1 10 50 100
LDLR
β-Gal
BSA
 0 1 10 50 100
β-Actin
LDL (ug/ml)
 0 1 10 50 100
β-Actin
LDLR
β-Gal
BSA
LDL (ug/ml)
 0 1 10 50 100
Same blots
Same blots
Re-probed for β-Actin
Re-probed for β-Actin

## Slide 3
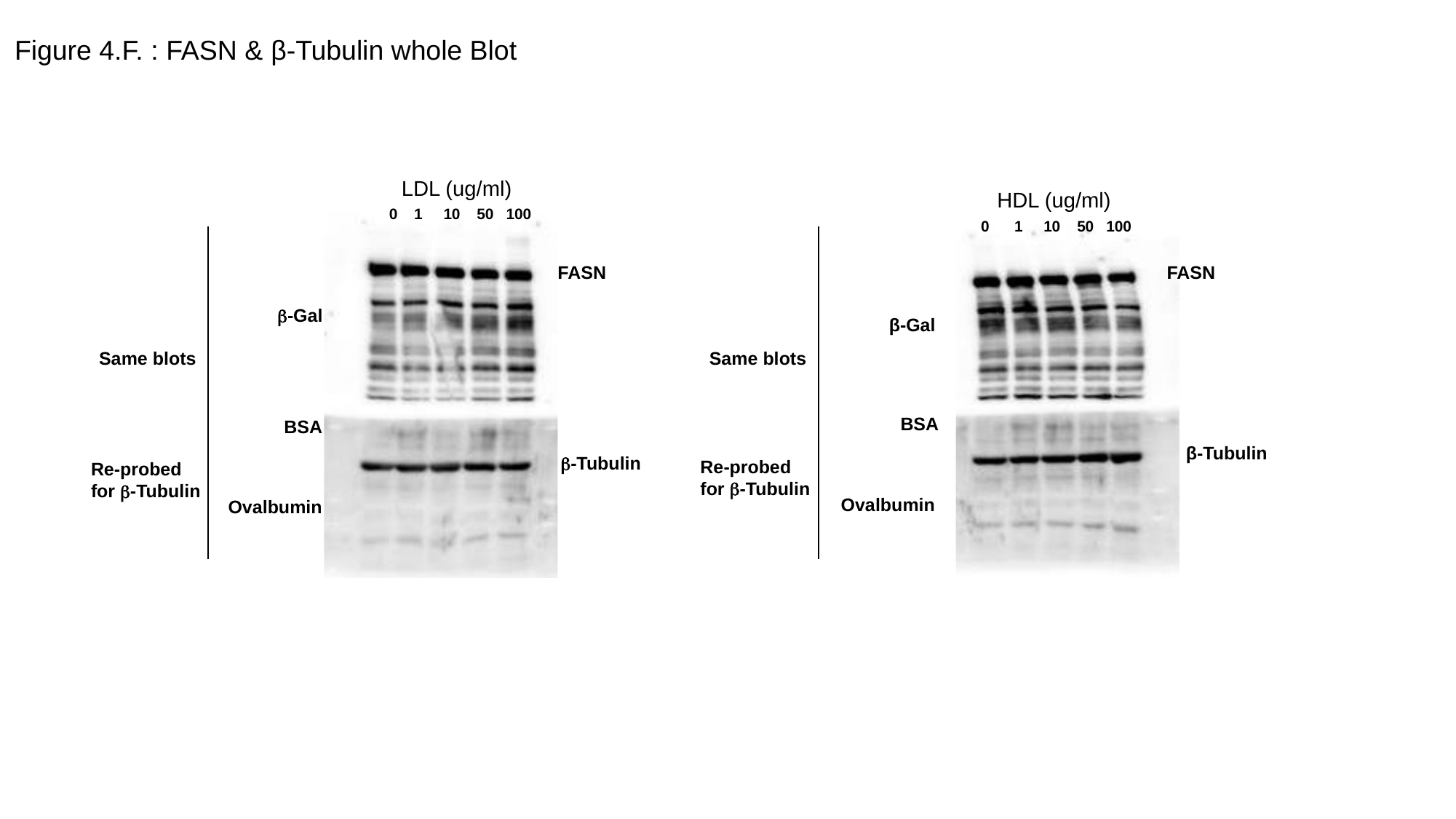

Figure 4.F. : FASN & β-Tubulin whole Blot
LDL (ug/ml)
0 1 10 50 100
FASN
b-Gal
BSA
b-Tubulin
Ovalbumin
HDL (ug/ml)
0 1 10 50 100
FASN
β-Gal
BSA
β-Tubulin
Ovalbumin
Same blots
Same blots
Re-probed for b-Tubulin
Re-probed for b-Tubulin

## Slide 4
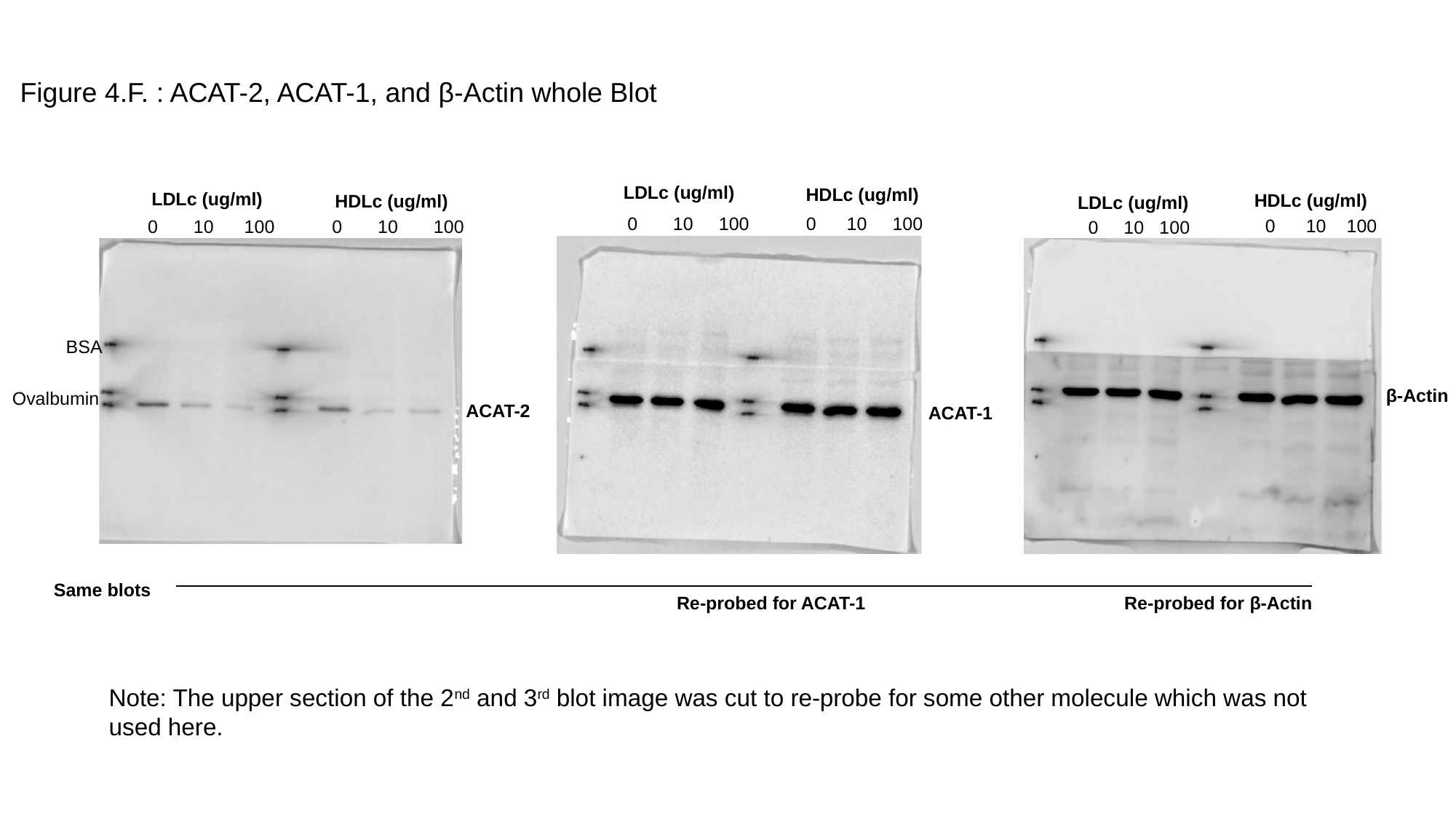

Figure 4.F. : ACAT-2, ACAT-1, and β-Actin whole Blot
LDLc (ug/ml)
HDLc (ug/ml)
 0 10 100
 0 10 100
ACAT-1
LDLc (ug/ml)
 0 10 100
BSA
Ovalbumin
ACAT-2
HDLc (ug/ml)
 0 10 100
HDLc (ug/ml)
LDLc (ug/ml)
 0 10 100
 0 10 100
β-Actin
Same blots
Re-probed for ACAT-1
Re-probed for β-Actin
Note: The upper section of the 2nd and 3rd blot image was cut to re-probe for some other molecule which was not used here.

## Slide 5
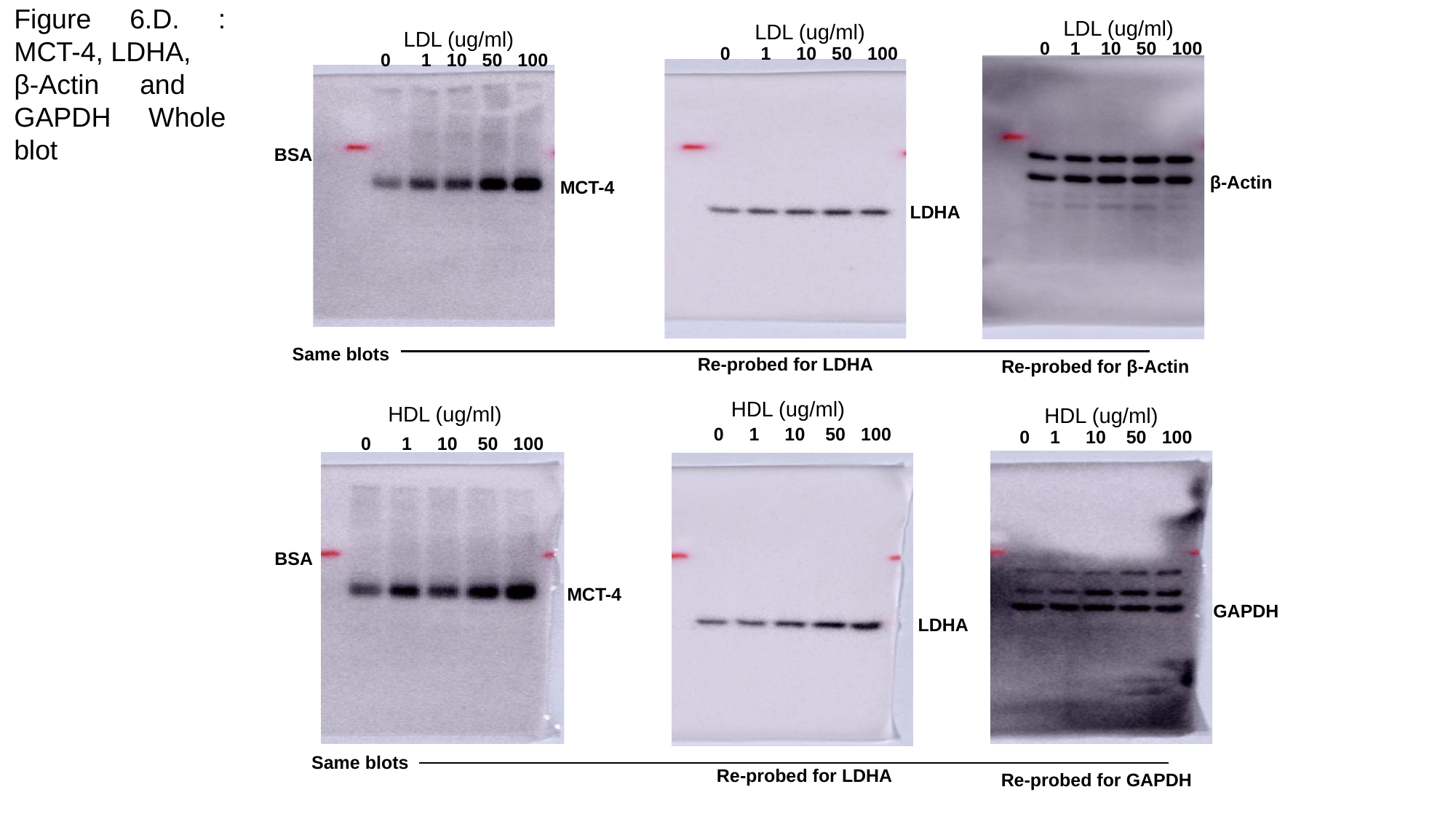

Figure 6.D. : MCT-4, LDHA,
β-Actin and GAPDH Whole blot
LDL (ug/ml)
LDL (ug/ml)
LDL (ug/ml)
0 1 10 50 100
0 1 10 50 100
0 1 10 50 100
MCT-4
LDHA
BSA
β-Actin
Same blots
Re-probed for LDHA
Re-probed for β-Actin
HDL (ug/ml)
HDL (ug/ml)
HDL (ug/ml)
0 1 10 50 100
0 1 10 50 100
0 1 10 50 100
MCT-4
BSA
LDHA
GAPDH
Same blots
Re-probed for LDHA
Re-probed for GAPDH

## Slide 6
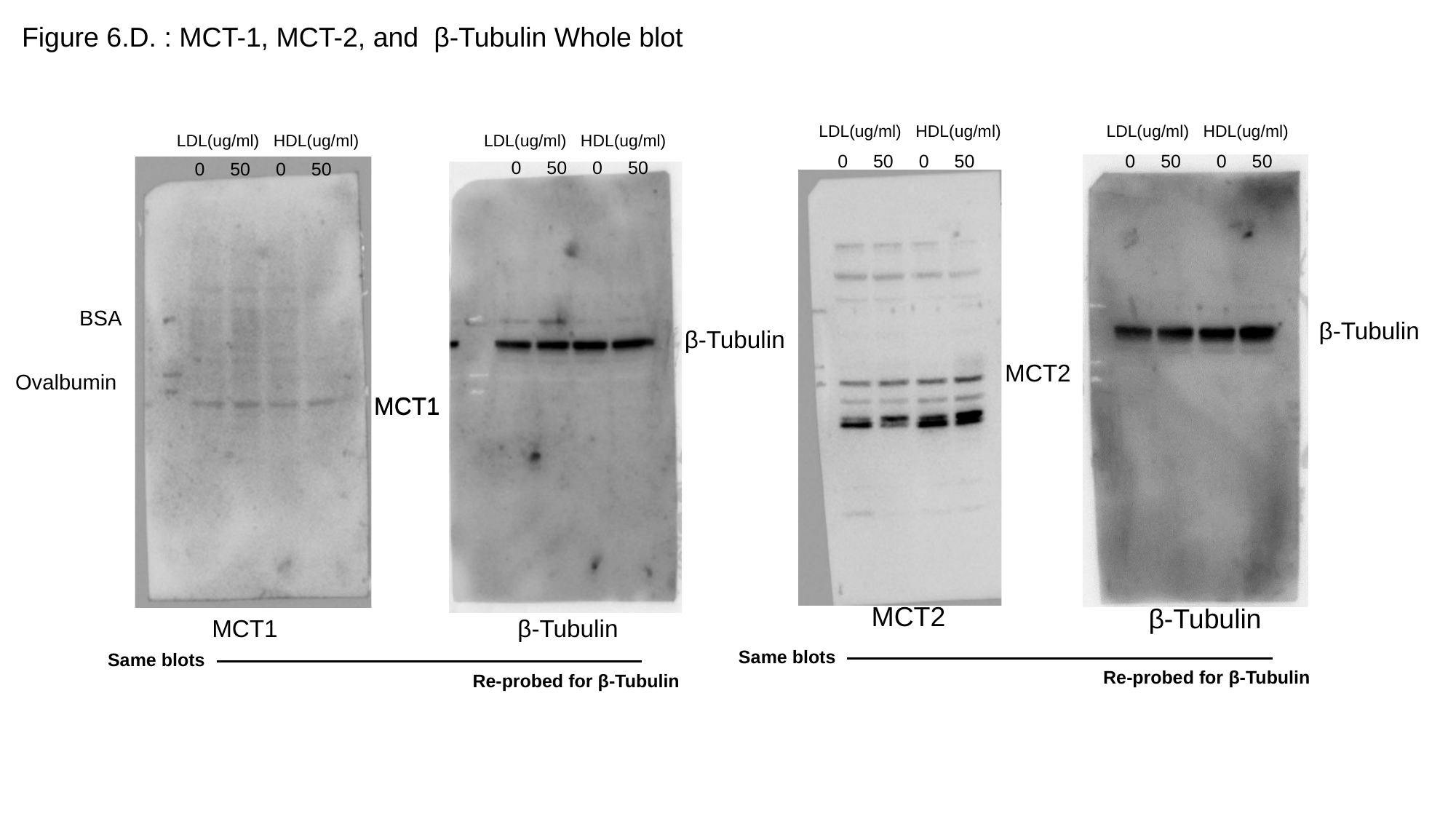

Figure 6.D. : MCT-1, MCT-2, and β-Tubulin Whole blot
 LDL(ug/ml) HDL(ug/ml)
 0 50 0 50
β-Tubulin
β-Tubulin
 LDL(ug/ml) HDL(ug/ml)
 0 50 0 50
MCT2
MCT2
 LDL(ug/ml) HDL(ug/ml)
 0 50 0 50
MCT1
MCT1
MCT1
 LDL(ug/ml) HDL(ug/ml)
 0 50 0 50
β-Tubulin
β-Tubulin
BSA
Ovalbumin
Same blots
Same blots
Re-probed for β-Tubulin
Re-probed for β-Tubulin
